# Regulatory Network Analysis of Mutated Genes Based on Multi-Omics Data Reveals the Exclusive Features in Tumor Immune Microenvironment Between Left-Sided and Right-Sided Colon Cancer

**DOI:** 10.3389/fonc.2021.685515

**Published:** 2021-06-15

**Authors:** Tianfei Yi, Yuwei Zhang, Derry Minyao Ng, Yang Xi, Meng Ye, Lvjun Cen, Jianjiong Li, Xiaoxiang Fan, Yanguo Li, Shiyun Hu, Hao Rong, Yangyang Xie, Guofang Zhao, Leyi Chen, Chen Chen, Shujing Ni, Jiaying Mi, Xiaoyu Dai, Qi Liao

**Affiliations:** ^1^ The Affiliated Hospital of Medical School of Ningbo University, Ningbo, China; ^2^ Department of Preventative Medicine, Zhejiang Provincial Key Laboratory of Pathophysiology, School of Medicine, Ningbo University, Ningbo, China; ^3^ Department of Biochemistry and Molecular Biology, Zhejiang Provincial Key Laboratory of Pathophysiology, School of Medicine, Ningbo University, Ningbo, China; ^4^ Hua Mei Hospital, University of Chinese Academy of Sciences, Ningbo, China; ^5^ Ningbo Institute of Life and Health Industry, University of Chinese Academy of Sciences, Ningbo, China; ^6^ Key Laboratory of Diagnosis and Treatment of Digestive System Tumors of Zhejiang Province, Ningbo, China; ^7^ Institute of Drug Discovery Technology, Ningbo University, Ningbo, China

**Keywords:** left-sided colon cancer (LCC), right-sided colon cancer (RCC), tumor immune microenvironment (TIME), gene mutation, multi-omics, immunotherapy, miRNA, methylation

## Abstract

Left-sided colon cancer (LCC) and right-sided colon cancer (RCC) have distinct characteristics in tumor immune microenvironment (TIME). Although existing studies have shown a strong association between gene mutations and TIME, whether the regulatory mechanisms between gene mutations and TIME are different between RCC and LCC is still unclear. In this study, we showed the fractions of CD8+ T cells were higher while those of regulatory T cells were lower in RCC. Besides, a stronger association between gene mutations and TIME was observed in RCC. Specifically, using multi-omics data, we demonstrated the mutations of most top mutated genes (TMGs) including *BRAF, PCLO, MUC16, LRP2, ANK3, KMT2D, RYR2* made great contributions to elevated fraction of immune cells by up-regulating immune-related genes directly or indirectly through miRNA and DNA methylation, whereas the effects of *APC, TP53* and *KRAS* mutations on TIME were reversed in RCC. Remarkably, we found the expression levels of several immune checkpoint molecules such as *PD-1* and *LAG3* were correlated with corresponding DNA methylation levels, which were associated with the mutations of TMGs in RCC. In contrast, the associations between gene mutations and TIME were less significant in LCC. Besides, survival analyses showed *APC* mutation had adverse impact on immunotherapy while patients with *BRAF* mutation were more suitable for immunotherapy in colon cancer. We hope that our results will provide a deeper insight into the sophisticated mechanism underlying the regulation between mutations and TIME, and thus boost the discovery of differential immunotherapeutic strategies for RCC and LCC.

## Introduction

Colon cancer (CC) is the third leading cause of cancer and the second most common cause of cancer-related deaths worldwide (1). Due to the wide application of colonoscopy, the incidence rate of CC has seen a decline in recent years. However, patients with CC are still faced with a high risk of disease recurrence, leading to a major cause of CC mortality (2). Fundamentally, the colon develops from two separate embryonic sections of primitive gut: the midgut, which develops into the cecum, ascending colon, hepatic flexure and proximal two-thirds of transverse colon, is defined as the right (proximal) colon; and the hindgut, which gives rise to the distal third of the transverse colon, splenic flexure, descending colon and sigmoid colon, and is defined as the left (distal) colon (3). Tumor development likewise differs depending on its colon location, therefore, CC is classified separately into left-sided colon cancer (LCC) and right-sided colon cancer (RCC), and are recognized as two different types of CC with distinct clinicopathological characteristics and molecular features (4). As reported, RCC patients tend to be older, mucinous, undifferentiated, and have shorter survival time compared to the LCC patients (5). Additionally, the preferred metastasis sites differ between patients with LCC and RCC too. LCC patients are prone to have liver and lung metastasis, while RCC patients tend to have peritoneal carcinomatosis (6).

In the last decade, biologists and bioinformaticians have been trying to understand the heterogeneity of the etiology underlying the distinct clinical manifestation between LCC and RCC based on their multi-omics data including gene expression, DNA methylation, mutation and so on. As an example, a comprehensive investigation of multi-omics data showed that RCC is more hypermethylated and more aggressive relative to LCC (7). Recently, another multi-omics study revealed crucial crosstalk between calcium homeostasis and immune/GPCR signaling process was found only in LCC (8). However, the interplays among different kinds of biomolecular events are not well studied to date.

Tumor immune microenvironment (TIME), which includes immune cells and immune-related molecules in a tumor microenvironment, is an important factor to consider when trying to determine the choice of treatment strategies and to form reliable prognosis biomarkers for cancer (9, 10). Previous studies have shown that the degree of immune infiltration in RCC is higher than that in LCC (11, 12), which may be a major contributor to the differences of drug-sensitivity and prognosis. Tumor immunity is often regulated by genetic and epigenetic alternation. For example, the down-regulation of *SLC64A* in RCC results in the loss of immunosuppressive capacity (8). Meanwhile, the *TP53* mutation often plays a negative role in tumor immunity in colon cancer and gastric cancer but reversely in breast and lung cancer (13). Furthermore, CC patients with both high tumor mutation burden (TMB) and *BRAF* mutation always present a high level of immune infiltration with an abundant expression of immune checkpoint molecules such as *PD-L1 (* 14), and *BRAF* is a biomarker for immunotherapy in melanoma (15). Additional, immune infiltration-associated lncRNAs can also act as biomarkers for immunotherapy such as *LINC01184 (* 16) and *LINC02256 (* 17) et al. However, whether the regulatory mechanisms between gene mutation and TIME are different between RCC and LCC remains unclear.

In this study, we performed a systematic association analysis on the top mutated genes (TMGs) and TIME in both types of CC and constructed the TMG-associated regulatory networks based on multi-omics data, which includes gene and miRNA expression, DNA mutation and methylation, to identify the potential molecular mechanisms behind these phenotypic differences. Furthermore, we explored the effects of *APC*, *BRAF* and *TP53* mutation on immunotherapy for CC patients. Our results will help us to better understand the tumorigeneses mechanisms of RCC and LCC, and discover new molecular prognostic and therapeutic targets.

## Materials and Methods

### Collection of Multi-Omics Data of CC Patients

All the multi-omics data of CC patients, including DNA mutation, gene expression, miRNA expression, DNA methylation, and clinical characteristics, were collected from The Cancer Genome Atlas (TCGA, https://cancergenome.nih.gov/) portal. Primary tumors located in the appendix to the transverse colon are defined as RCC, whereas tumors located from the splenic flexure to the sigmoid colon are categorized as LCC. 1134 CC patients with gene mutations were selected from the cBioPortal database, in which 1085 patients with prognosis and location information were used for survival analysis. Of these patients, 80 (37 RCC and 43 LCC) were treated with immunotherapy (https://www.cbioportal.org/).

For DNA mutation, we collected the Mutation Annotation Format (MAF) files that record the information of somatic variants processed from whole-exome sequencing data of 322 CC patients. For gene expression, the expression profiles of 428 CC samples and 39 normal samples were obtained. For miRNA, the expression profiles of 407 CC samples and 8 normal samples were obtained from the small RNA-seq data. For DNA methylation, we collected the beta values of 272 CC samples and 35 normal samples, which represent the ratio of the methylated probe intensity and total intensity detected from the Illumina HumanMethylation450 BeadChip Arrays. The clinicopathologic features such as age, gender, cancer stage, TNM stage, and survival data for 434 CC patients were also collected. The sample sizes for each type of dataset in the LCC and RCC cohort were shown in **Supplementary Table 1**.

### Molecular Subtypes of CC

Microsatellite Instability (MSI) and chromosomal instability (CIN) subtypes information was downloaded from the cBioPortal database (https://www.cbioportal.org/). Among them, the samples were divided into three groups levels of MSI, denoted as microsatellite instability-high (MSI-H), microsatellite instability-low (MSI-L), and microsatellite stability (MSS) respectively. While CIN was classified into CIN and non-CIN. CpG island methylator phenotype (CIMP) was determined by the DNA methylation level of five markers (*CACNA1G*, *IGF2*, *NEUROG1*, *RUNX3*, and *SOCS1*) using a similar method as previously reported (18). In brief, the intensities of 307 CpG sites within the five genes were extracted from the DNA methylation profiles and used for unsupervised consensus clustering analysis. To confirm the superiority of CIMP subtype classification based on these five genes, we compared the results with those based on 7 genes of DNA methyl-transferases (*DNMT1, DNMT3A, DNMT3B* and *DNMT3L*) and demethylases (*TET1, TET2* and *TET3*), which were well-acknowledged DNA methylation-associated genes. According to the clustering result, the CIMP phenotype can be divided into two categories, high level of CIMP (CIMP-H) and low level of CIMP (CIMP-L).

### Evaluation of TIME for CC Patients

To evaluate the infiltration level of immune and stromal cells infiltration, we used ESTIMATE (Estimation of Stromal and Immune cells in Malignant Tumor tissues using Expression data) algorithm (19) to calculate the immune and stromal score for each sample using the expression profile, in which a higher immune/stromal score would represent a higher degree of immune/stromal cells filtration. The fractions for 22 immune cells were obtained from the expression profiles using CIBERSORTx software (20). The samples with *p*-value <0.05 were considered significant and selected for analyses. To further compare the proportions of different types of T cells between the two cohorts, ImmuCellAI was employed to evaluate the abundance of 18 T cells (21). Wilcoxon test was used to compare the difference of immune cell proportions between LCC and RCC.

### Association Analysis of Clinical Features

For the association analysis of the clinical features and location, *t*-test was used to compare two groups of measurement data, Pearson’s *chi*-squared test was applied for categorical data while Mantel-Haenszel’s *chi*-squared test was used to compare two groups of ordinal data. For association analyses of the clinical features and immune score, Wilcoxon test was used to compare two groups while Kruskal Wallis H test was applied for multiple groups. The accepted level of significance is *p*<0.05. Kaplan–Meier survival curves were performed for prognostic differences and compared using the Tarone-Ware test.

### TMGs Identification, Mutated Pairs Characterization, and Visualization

The analysis of the MAF files was conducted using the ‘maftools’ R package, which has various analytical and visualization methods that are commonly used in cancer genomic studies (22). Next, we obtained the mutation rate of each gene in patients with LCC and RCC respectively. The genes were sorted in descending order by the mutation rates, and the top 30 frequently mutated genes were obtained and defined as TMGs in each cohort. Furthermore, the mutated pairs of TMGs in a mutually exclusive or co-occurring manner were characterized using the Fisher’s exact test on a 2×2 contingency table containing the frequencies of mutated and non-mutated samples. The *p*-values were adjusted using the false discovery rate (FDR) method. FDR<0.05 was a cutoff to select significant pairs. The TMGs associated networks were visualized using the Cytoscape software.

### Tumor Mutation Burden Calculation

For each sample, we counted the number of somatic variants occurring throughout the coding regions of the sequenced gene and defined it as the somatic mutation frequency (SMF). Next, we normalized SMF per 30 megabase pairs (Mbp) and divided it by the total genomic territory sequenced. Therefore, TMB was calculated using the following formula:

$$TMB=\frac{SMF}{30Mbp}$$

### Differential Expression Analysis of Genes and miRNAs

For gene expression, we first removed the low expressed genes with an average FPKM less than 0.1 or the samples with more than 20% of genes lacking expression values. Next, a differential expression analysis between cancer samples and corresponding normal samples was performed in RCC and LCC respectively using the ‘DESeq2’ R package. The genes with FDR<0.05 and |log2(Fold Change (FC)) | >log2(1.5) in each cohort were considered as the differentially expressed genes (DEGs). As for miRNA, we removed the miRNAs with low or absent expression and obtained the differentially expressed miRNAs (DEmiRNAs) in RCC or LCC by using the same method and cutoff as above-mentioned.

### Differential Methylation Analysis

The processing of DNA methylation profiles was conducted using the Chip Analysis Methylation Pipeline (ChAMP) R package (23) in RCC and LCC respectively. First, the probes with more than 20% of samples lacking signals and the samples with more than 10% of probes lacking values were removed, in total, 395413 (81.4%) probes and 320 (100%) samples were eligible for further analysis. The normalization of probe signals was performed using peak-based correction (PBC) method (24). The probes with both FDR<0.05 and beta-value difference (△ß)>0.15 were considered as differentially methylated probes (DMPs).

### Construction of TMG-Centric Regulatory Network

For each TMG, we divided the CC patients into two groups: mutation carriers and wildtype (non-mutation) carriers. Then, *t*-test was applied to reveal the TMG-associated DEGs by comparing the TMG-mutated and TMG-wildtype group in each cohort, and *P*-values were adjusted using the FDR method. The genes with FDR<0.05 and Log2(FC)>Log2(1.5) were defined as the TMG-associated genes (i.e., TMG-DEG pairs). After which, we applied the same method to identify the TMG-associated miRNAs (i.e., TMG-DEmiRNA pairs). To obtain TMG-associated DNA methylation probes, we used the *t*-test to determine whether the DNA methylation level of each probe was significantly different between the mutation and wildtype group for each TMG. The probes with FDR<0.05 and △ß >0.15 were defined as the TMG-associated probes (i.e., TMG-DMP pairs). Finally, the TMG-centric regulatory network was created using TMG-DEG, TMG-DEmiRNA, and TMG-DMP relationships, which were divided into two parts: 1) the negative relationships, i.e., the levels of the DEGs, DEmiRNAs, or DMPs in mutation carriers are lower than those in non-mutation carriers, which indicated that TMGs regulate these molecules negatively; 2) the positive relationships, where the regulation are in the reverse direction. Network visualization was performed using Cytoscape software.

### miRNA-Target Relationships Prediction

We obtained the predicted miRNA-target relationships by combining the results from two sources: predicted using miRANDA software with default parameters and data that was directly downloaded from the miRDB database. Due to the inhibitory effect of miRNA on gene expression, we finally retained miRNA-target pairs with opposite differential expression trends for downstream analysis.

### Functional Enrichment Analysis

GO functional enrichment analysis was performed by “ClusterProfiler” R package. The terms with FDR<0.05 and the gene number corresponding to this term not less than 3 were considered to be statistically significant.

### Selection of Immune-Related GO BP Terms

The terms with the following keywords were defined as immune-related BPs: ‘interferon-gamma’, ‘immunity’, ‘immune’, ‘cytokine’, ‘T cell’, ‘B cell’, ‘neutrophil’, ‘leukocyte’, ‘macrophage’, ‘T-helper’, ‘interleukin’, ‘chemotaxis’, ‘chemokine’, ‘inflammatory’, ‘natural killer’, ‘eosinophil’, ‘lymphocyte’. The TMGs or miRNAs with at least one immune-related BP term were considered as immune-related TMGs or miRNAs.

### Identification of Immune-Related Genes, miRNAs and Methylation Probes Globally

First, the CC patients in LCC and RCC cohort were classified into either the high-immunity group (immune score>=median) or the low-immunity group (immune score<median) based on the median value of their immune score in each cohort. The significant immune-related DEGs, DEmiRNAs and DMPs were determined by *t*-test between high-immunity and low-immunity groups with thresholds of FDR<0.05 and |log2(FC)| > |log2(1.5)|. The significant immune-related DEGs with log2(FC)>0 were considered as immune-promoting molecules, indicating these molecules are up-regulated in the high-immunity group compared to the low-immunity group. While the significant immune-related DEGs with log2(FC)<0 were thought as immunosuppressive molecules, meaning these molecules are down-regulated in the high-immunity group compared to the low-immunity group. Finally, by taking the intersection for the TMG association network (including TMG-DEG, TMG-DEmiRNA, TMG-DMP) obtained from the previous analysis and the significant immune-related molecules (DEGs, DmiRNAs, DMPs), we obtained immune regulatory network. Network visualization was performed using Cytoscape software.

## Results

### Clinical Features and Tumor Immune Microenvironment of RCC and LCC Patients

A total of 434 CC patients from the TCGA-COAD portal were divided into LCC group (n=176) and RCC group (n=258) according to the location of their primary tumor. Then, we compared the clinical characteristics between the two cohorts. In line with existing research (25), RCC patients tend to be older and have a worse overall prognosis compared to LCC patients (**Table 1** and **Figure S1**). Next, we analyzed the associations between the location and other kinds of molecular subtype including microsatellite instability (MSI), chromosomal instability (CIN), and CpG island methylator phenotype (CIMP) which are classified based on their distinct genomic and molecular characteristics. Consistent with existing study (26), we also found that RCC group has a higher proportion of MSI-H and a lower proportion of CIN patients compared to LCC group (**Figures S2A, B** and **Table 1**). As for CIMP, we determined the CIMP subtype for each patient using the β value of DNA methylation panel or 7 methylation-related genes respectively. The DNA methylation panel, which consists of five markers, *CACNA1G, IGF2, NEUROG1, RUNX3*, and *SOCS1* (18) showed CIMP distribution of CC patients well. However, the 7 genes of DNA methyl-transferases or demethylases, including *DNMT1, DNMT3A, DNMT3B, DNMT3L, TET1, TET2* and *TET3*, showed poor CIMP clustering result (**Figure S3B**). Therefore, we distinguished CIMP subtypes based on the above five markers. The results showed that RCC group had a higher proportion of CIMP-H patients (**Figures S2C**, **S3A** and **Table 1**).

**Table 1:** Comparison of molecular subtypes and clinical characteristics between RCC and LCC (N =434).

Characteristics	Category	RCC(N/%)	LCC(N/%)	T-test/*χ* ^2^	*P*
Microsatellite	MSI-H^#^	72(28.5)	4(2.4)	35.352^a^	<0.001*
	MSI-L^#^	39(15.4)	34(20.7)		
	MSS^#^	142(56.1)	126(76.8)		
Methylation	CIMP-H^#^	94(54.7)	4(4.0)	70.388^b^	<0.001
	CIMP-L^#^	78(45.3)	96(96.0)		
Chromosome	CIN^#^	107(43.0)	104(63.8)	17.110^b^	<0.001*
	Non-CIN^#^	142(57.0)	59(36.2)		
Age		68.51 ± 13.37	64.89 ± 12.48	2.848^c^	0.005*
Stage	I	44(17.7)	30(17.2)	2.862^a^	0.091
	II	109(43.8)	62(35.6)		
	III	68(27.3)	51(29.3)		
	IV	28(11.2)	31(17.8)		
Gender	Man	135(52.3)	92(52.3)	0^b^	1
	Woman	123(47.7)	84(47.7)		
Race	American Indian or Alaska Native	0(0)	1(1.0)	4.725^b^	0.193
	Asian	8(4.7)	3(3.2)		
	Black or African American	41(24.3)	15(16.0)		
	White	120(71.0)	75(79.8)		
Intestinal polyp	Yes	78(35.5)	55(35.7)	0.003^b^	0.959
	No	142(64.5)	99(64.3)		
Relapse	Yes	42(19.9)	34(23.6)	0.699^b^	0.403
	No	169(80.1)	110(76.4)		
Lymphatic invasion	Yes	83(35.9)	70(43.5)	2.271^b^	0.132
	No	148(64.1)	91(56.5)		
T	Tis^#^	1(0.4)	0(0)	1.477^a^	0.224
	T1^#^	5(1.9)	4(2.3)		
	T2^#^	42(16.3)	34(19.3)		
	T3^#^	174(67.4)	122(69.3)		
	T4^#^	36(14.0)	16(9.1)		
Metastases	M0^#^	194(87.4)	130(80.7)	3.159^b^	0.075
	M1^#^	28(12.6)	31(19.3)		
Node	N0^#^	163(63.2)	96(54.5)	0.565^a^	0.452
	N1^#^	45(17.4)	51(29.0)		
	N2^#^	50(19.4)	29(16.5)		

^a^Mantel-Haenszel’s chi-squared test; ^b^Pearson’s chi-squared test; ^c^T-test;

^#^MSI-H, Microsatellite instability-high; MSI-L, Microsatellite instability-low; MSS, Microsatellite stability;

CIMP-H, CpG island methylator phenotype-high; CIMP-L, CpG island methylator phenotype-low;

CIN, Chromosome instability; Non-CIN, Nonchromosomal instability;

Tis: In situ, non-invasive (confined to epithelium); T1: Small, minimally invasive within primary organ site; T2: Larger, more invasive within primary organ site; T3: Larger and/or invasive beyond margins of primary organ site; T4: Very large and/or very invasive, spread to adjacent organs;

M0: No distant metastases; M1: Distant metastases present;

N0: No lymph node involvement; N1: Regional lymph node involvement; N2: Extensive regional lymph node involvement;

*P<0.05.

**Figure 1:**
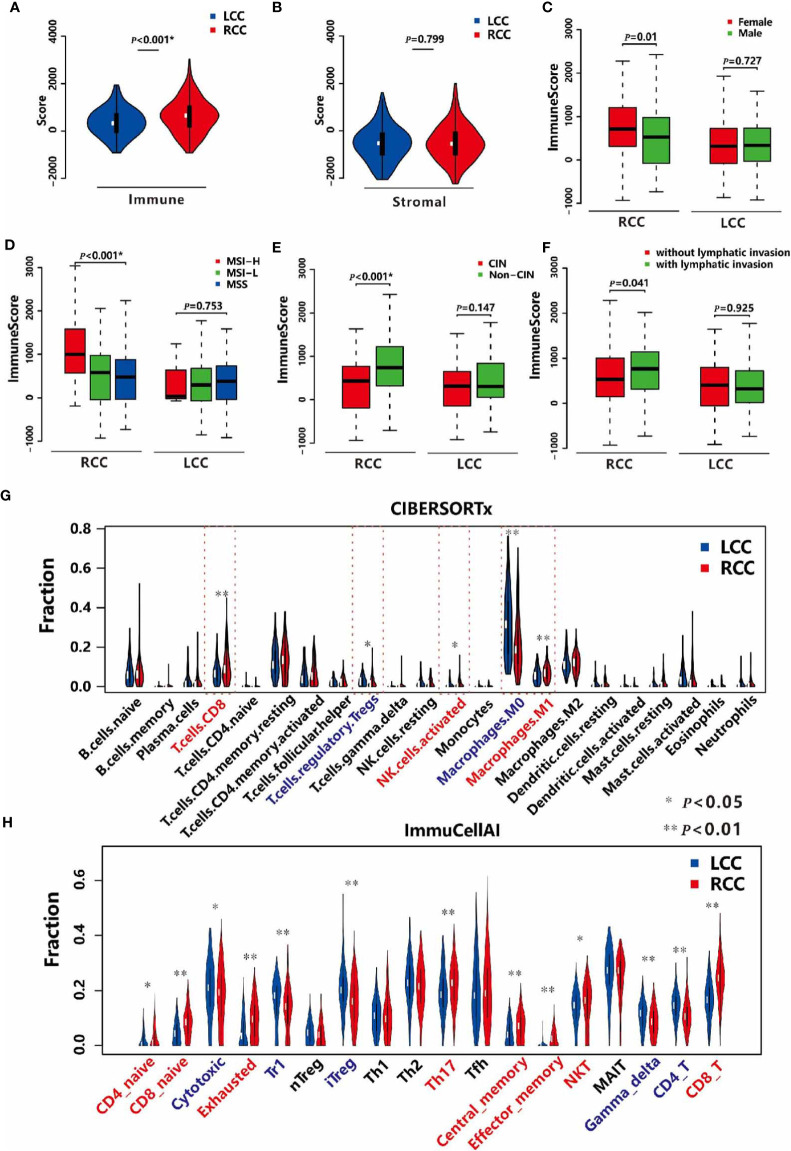
TIME in LCC and RCC. **(A)** Comparison of the immune scores between LCC and RCC. **(B)** Comparison of the stromal scores between LCC and RCC. **(C)** Comparison of the immune scores between female and male patients in RCC and LCC respectively. **(D)** Comparison of immune scores among patients with MSI-H, MSI-L, and MSS in RCC and LCC respectively. **(E)** Comparison of immune scores between patients with CIN and non-CIN in RCC and LCC respectively. **(F)** Comparison of immune scores between patients with and without lymphatic invasion in RCC and LCC respectively. **(G)** Comparison of 22 immune cells based on CIBERSORTx software between RCC and LCC. **(H)** Comparison of 18 T cells based on ImmuCellAI software between LCC and RCC.

TIME plays a crucial role in tumor progression (27). First, we obtained the immune and stromal scores using the ESTIMATE algorithm (19), which measures the levels of immune and stromal cells infiltration in samples respectively. Next, we compared the two kinds of score in cancerous tissues between LCC and RCC patients. We found the immune scores of cancer tissues in RCC were higher than those in LCC (**Figure 1A**). However, the differences in stromal scores were not significant (**Figure 1B**), suggesting that immune cells infiltration may play a specific role in tumor growth, prognosis and therapy for RCC. Then, the associations between the immune scores and the clinical factors in both types of CC were investigated. In RCC, the immune scores were significantly higher in patients with MSI and non-CIN subtypes (*P*<0.001, **Figures 1D, E**), and female patients had slightly higher immune scores than males’ (*P*=0.01, **Figure 1C**). However, there were no associations between immune scores and gender, MSI, and CIN in LCC (**Figures 1C–E**). Furthermore, the immune scores were slightly different between patients with and without lymphatic invasion in RCC (*P*=0.041) but not in LCC (**Figure 1F**). As the immune score is used to evaluate the overall level of immune cells infiltration, we estimated the fractions of 22 immune cells in detail to study the difference in TIME between LCC and RCC patients. As a result, we found that the fractions of CD8+ T cells, activated NK cells, and M1 Macrophages were significantly higher in RCC than LCC, while those of immune repressive cell types such as regulatory T cells, M0 Macrophages were significantly lower (**Figure 1G**). As T cells have many subsets with specific function, we further compared 18 T cell types between the two cohorts. As shown in **Figure 1H**, the proportions of CD4+ and CD8+ naïve, exhausted, Th17, central memory, effector memory, nature kill, and CD8+ T cells were all higher in the RCC group compared to the LCC group, while CD4+ T cells, cytotoxic, and regulatory T cells including Tr1 and iTreg, gamma delta were more abundant in LCC compared to RCC. These results revealed the different associations between TIME and molecular subtypes in LCC and RCC.

### Identification of TMGs in LCC and RCC

**Figure 2:**
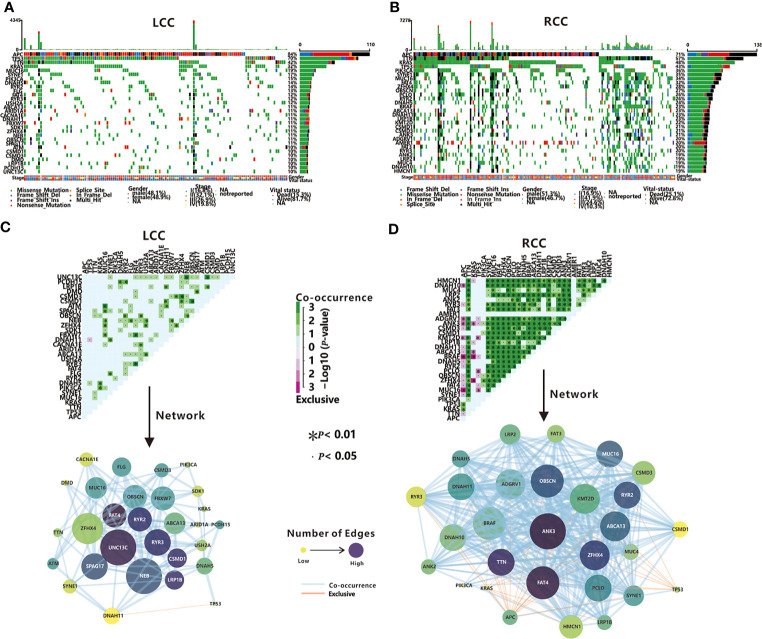
TMGs in LCC and RCC. Waterfall plots show the mutation status (middle)and the mutation rate (right)of each TMG, and the mutation frequency (top), the gender, stage and vital status (bottom) of each patient in LCC **(A)** and RCC **(B)**. Mutually exclusive and co-occurring gene pairs (top), and networks of mutational interaction (bottom) in LCC **(C)** and RCC **(D)**.

The exome sequencing data of 129 LCC patients and 193 RCC patients were used to identify TMGs. First, comparing with LCC, RCC patients have a higher TMB ($\bar{x}$ = 5.54 in LCC and $\bar{x}$ = 18.54 in RCC, *P*<0.05 by *t*-test), which was consistent with a previous report (28). Next, we selected the top 30 frequently mutated genes to be TMGs in each cohort. Among them, 18 are common, including some reported cancer-associated genes such as *APC*, *TP53*, *TTN*, *KRAS*, and *PIK3CA* (**Figures 2A, B** and **Supplementary Table 2**). As expected, most of these genes have higher mutation frequency in RCC compared to LCC, except for *APC* and *TP53* (84% vs. 71%, 70% vs. 57% in LCC and RCC respectively, **Figure S4**), suggesting the potential dominant roles of *APC* and *TP53* in LCC as previously shown (7).

Previous studies have found that the driver genes in dysregulated pathways are often mutated in a mutually exclusive manner, while co-mutated genes linked by synergism promote cancer development in a synergistic manner (29). Next, we investigated the interactions among TMGs in LCC and RCC respectively. We found that the *TP53*/*DNAH11* pair was the only mutually exclusive pair in LCC (**Figure 2C**). In contrast, 31 mutually exclusive pairs were identified in RCC (**Figure 2D**), of which *APC*, *TP53*, and *KRAS* were the most major exclusive genes and these three TMGs might play different roles from other TMGs in the initiation and progression of RCC. Remarkably, both *BRAF* and *ANK3* were mutually exclusive in all three genes (**Figure 2D**), which indicated that *BRAF* and *ANK3* may contribute greatly to the development of RCC through the involvement of multiple key pathways. Regarding the co-occurring pairs, *UNC13C*, *NEB*, and *ZFHX4* were the major co-occurring genes in LCC (**Figure 2C**), whereas a majority of TMGs in RCC interacted with each other in a co-occurrence manner (**Figure 2D**), further proving the complicated tumorigeneses mechanism of RCC. Unexpectedly, no interactions were found for *APC* in LCC and *AMER1* in RCC, suggesting they might have an independent role in LCC and RCC respectively. In summary, our results demonstrated that somatic interactions were significantly more frequent in RCC compared to LCC, which might be one of the factors underlying the poorer prognosis observed in RCC patients.

### Association Analysis of TMGs and TIME

**Figure 3:**
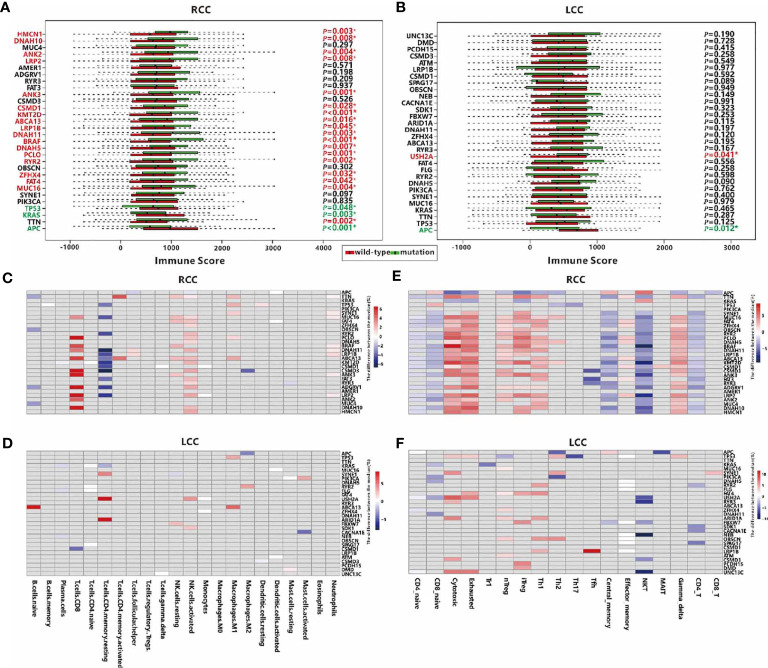
Associations between TMGs and TIME in RCC and LCC. Associations between mutation of TMGs and the immune score in RCC **(A)** and LCC **(B)** respectively. TMGs marked in red are associated with a higher immune score when they are mutated while TMGs marked in green have the opposite effect. Associations between TMGs and 22 immune cells using the Wilcoxon test in RCC **(C)** and LCC **(D)**. Associations between TMGs and 18 T cells using the Wilcoxon test in RCC **(E)** and LCC **(F)**. The grey cells represent insignificant associations while the colored cells represent significant associations with *p*<0.05. The red color represents a higher immune cell fraction in mutation group while the blue color represents a lower immune cell fraction.

Next, we explored the association between the mutation status of each TMG and TIME in each cohort. In RCC, mutations of 20 TMGs (20/30, 66.7%) were observed to be related to the immune score, and that patients with mutations in most of TMGs have a higher immune score, except for the mutations of *TP53*, *APC*, and *KRAS* (**Figure 3A**). As previously stated, we found that *TP53*, *APC*, and *KRAS* were main mutually exclusive TMGs in RCC (**Figure 2D**), suggesting the three genes have different roles on TIME in RCC. However, only 2 TMGs (*APC* and *USH2A*, 2/30, 6.7%) were associated with the immune score in LCC (**Figure 3B**), suggesting TMGs in RCC have a greater impact on TIME compared to LCC. Strikingly, a positive trend of immune score, although not significant (*P*=0.125), was observed in LCC patients with *TP53* mutation (**Figure 3B**), which was contrary to its effect in RCC.

To better understand the association between TMGs and TIME, we studied the relationships between the mutation status of each TMG and the fractions of 22 immune cells or 18 T cells. In RCC, 27 TMGs were found to be associated with at least one type of immune cell (**Figure 3C**), and 28 TMGs were associated with at least one type of T cell (**Figure 3E**). For example, the infiltration of CD8+ T cell was positively correlated with 11 TMGs such as *PCLO, CSMD3, ANK3* and *LRP2* et al., while that of resting CD4 memory T cell was negatively correlated with 12 TMGs such as *DNAH11, KMT2D, CSMD3* and *LRP2* et al. (**Supplementary Table 3**). Besides, we found that the proportions of cytotoxic, exhausted, nTreg, iTreg, Th1 and gamma delta cells were positively correlated with 11 TMGs, such as *TTN, MUC16, FAT4, BRAF* et al., while those of CD8+ naïve, central memory, NKT and CD4+ T cells were negatively correlated with 17 TMGs, such as *MUC16*, *FAT4*, *DNAH11*, *BRAF* et al. In LCC, only 22 TMGs were found to be associated with at least one type of immune cell (**Figure 3D**), and 26 TMGs were associated with at least one type of T cell (**Figure 3F**). For example, the fraction of resting CD4 memory T cell was positively correlated with *USH2A* and *ARID1A* mutation, while that of CD8+ T cell was negatively correlated with *CSMD1* mutation (**Supplementary Table 3**). In addition, we also observed the relationships between 18 T cell types and TMGs in LCC were significantly poorer than those in RCC. Obviously, the fractions of cytotoxic, exhausted, nTreg, iTreg and Th1 cells were positively correlated with partial TMGs, while those of NKT, CD4+ T cells and CD8+ naïve T cells were negatively correlated with some TMGs (**Supplementary Table 3**). In conclusion, TMGs may play a crucial role in RCC TIME.

### TMGs Influence TIME Through Regulation of Gene Expression

**Figure 4:**
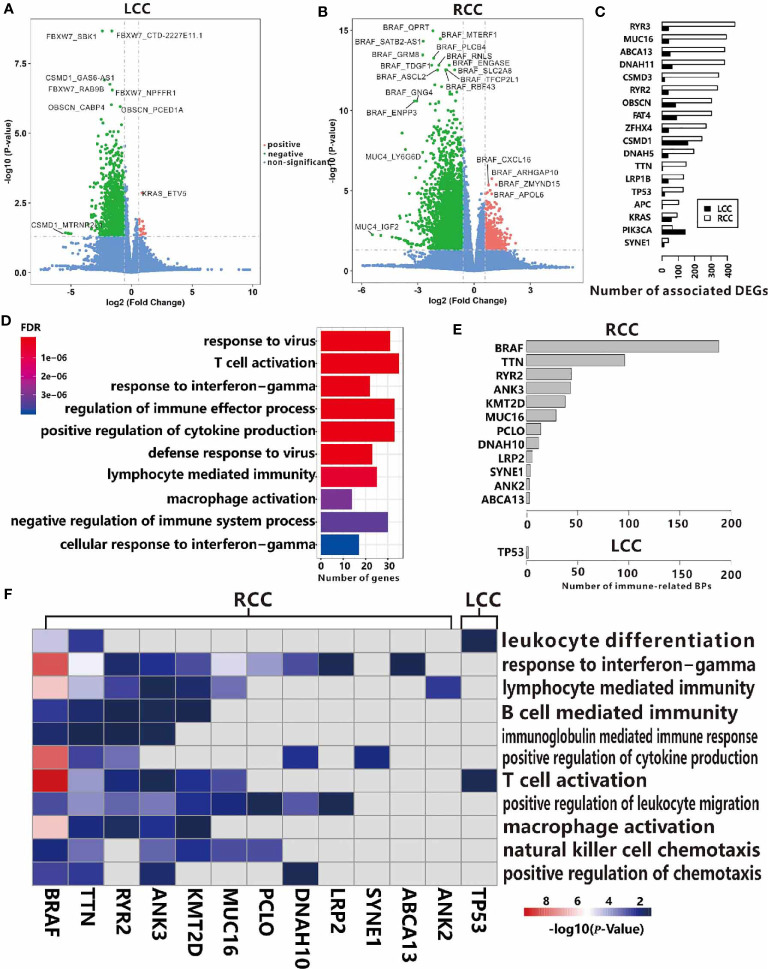
Associations between TMGs and DEGs in RCC **(A)** and LCC **(B)**. **(C)** The number of associated DEGs of the 18 common TMGs in RCC and LCC. **(D)** GO functional enrichment analysis of TMG-upregulating DEGs in RCC. **(E)** The number of immune-related BP in the up-regulated genes for each TMG in RCC and LCC. **(F)** The heatmap showing the -log10(*P*-values) of immune-related BP. The color strength represents the -log10(*P*-values) of associations, the grey cells represent insignificant associations in RCC and LCC.

To illustrate the distinct regulatory mechanisms of TMGs influencing TIME, we further explored the genes whose expression levels were associated with TMGs in the two types of CC. Based on the RNA-seq profiles of 256 RCC and 172 LCC patients from the TCGA portal, DEGs were identified by comparing the expression values of cancer samples to the corresponding normal control tissues. With the threshold of the adjusted p-values < 0.05 and fold change (FC) > 1.5, 4694 up-regulated and 3596 down-regulated genes were identified in LCC, while 4882 up-regulated and 3505 down-regulated genes were found in RCC. Next, we sought to investigate the potential associations between TMGs and the two sets of DEGs respectively. For each TMG in LCC or RCC, we compared the expression level of the DEGs between the TMG-mutation and the TMG-wildtype groups using *t-*test. We identified 19 positive (higher expression of DEG in mutation group) and 2084 negative (lower expression of DEG in mutation group) TMG-DEG pairs in LCC (**Figure 4A** and **Supplementary Table 4**), while 1131 positive and 8189 negative TMG-DEG pairs in RCC (**Figure 4B** and **Supplementary Table 4**). Among them, 88 TMG-DEG pairs were common in both LCC and RCC. The number of TMG-DEG pairs in RCC is much higher compared to LCC, suggesting a stronger relationship between TMG and gene expression regulation in RCC. Furthermore, even the common TMGs between LCC and RCC have a different number of associated genes (**Figure 4C**), further hinting that the regulatory network of TMGs is different between LCC and RCC. We then attempted to find the TMGs associated processes that were altered in LCC and RCC, we performed functional enrichment analysis on the TMG-upregulating and TMG-downregulating genes in both types of CC. The results showed that there were 464 TMG-upregulating genes in RCC, and they were mainly associated with the immune system including T cell activation, regulation of immune effector process, and positive regulation of cytokine production (**Figure 4D**), while TMG-downregulating genes involved with the genes that were related to various metabolic, blood and heat-related processes such as steroid metabolic process, cellular hormone metabolic process, regulation of blood pressure, heat generation, etc. (**Figure S5A**). As for LCC, TMG-downregulating genes showed significant enrichment of terms associated with muscle contraction, chondrocyte differentiation, cartilage development, etc. (**Figure S5B**), and no enrichment results were found in the TMG-upregulating genes, which was likely because only 19 genes were involved. To further explore the functions of DEGs positively regulated by TMG mutations in LCC, we picked the top 1000 genes with highest FC to perform functional enrichment analysis (**Figure S5C**), finding that these genes were also related to the humoral immune response. The results revealed that mutations of TMGs can up-regulate immune-related genes in both LCC and RCC, but the effect is much wider and stronger in RCC than in LCC.

The results from a global association analysis of TMGs mutation and TIME led us to speculate that some TMGs may have a strong association with TIME and this relationship may be different between LCC and RCC. To link TMGs to specific immune processes, we conducted functional enrichment analyses for the up-regulated genes and down-regulated genes of each TMG in LCC and RCC respectively. We found that 12 TMGs in RCC and only *TP53* in LCC were associated with at least one immune-related process based on the enrichment results of corresponding up-regulated genes for each TMG (**Figures 4E, F** and **Supplementary Table 5**). However, based on the enrichment results from the down-regulated genes of each TMG, no TMG was found to be associated with the immune-related process in both LCC and RCC. These results further indicated that more TMGs in RCC were related to an elevated expression level of immune-related genes. For example, *BRAF* had the most associated genes in RCC. We found that 310 up-regulated genes associated with *BRAF* mutation were enriched in the genes related to T cell activation, response to interferon-gamma, positive regulation of cytokine production, and other immune-related processes (**Figure S6A**). While its 637 down-regulating genes were mainly involved in neuron recognition, negative regulation of the Wnt signaling pathway, lipid transport, etc. (**Figure S6B**). Some associated genes of *BRAF* mutation had been reported to have important functions in CC. For instance, lncRNA *SATB2-AS1* was found to be up-regulated when *BRAF* mutation occurs in RCC in our study, and it was recently reported to inhibit tumor metastasis and affect tumor immunity in CC (30), indicating that *BRAF* plays a crucial role in TIME. Surprisingly, we found that *TP53* mutations were positively associated with 11 genes in LCC, which were enriched with the genes relating to T cell activation and leukocyte differentiation (**Figure 4F**). Considering the negative role of *TP53* to TIME in RCC as shown above (**Figure 3A**), we hypothesized that *TP53* may play a positive role on TIME only in LCC.

### TMGs Influence TIME Through Regulation of miRNAs

**Figure 5:**
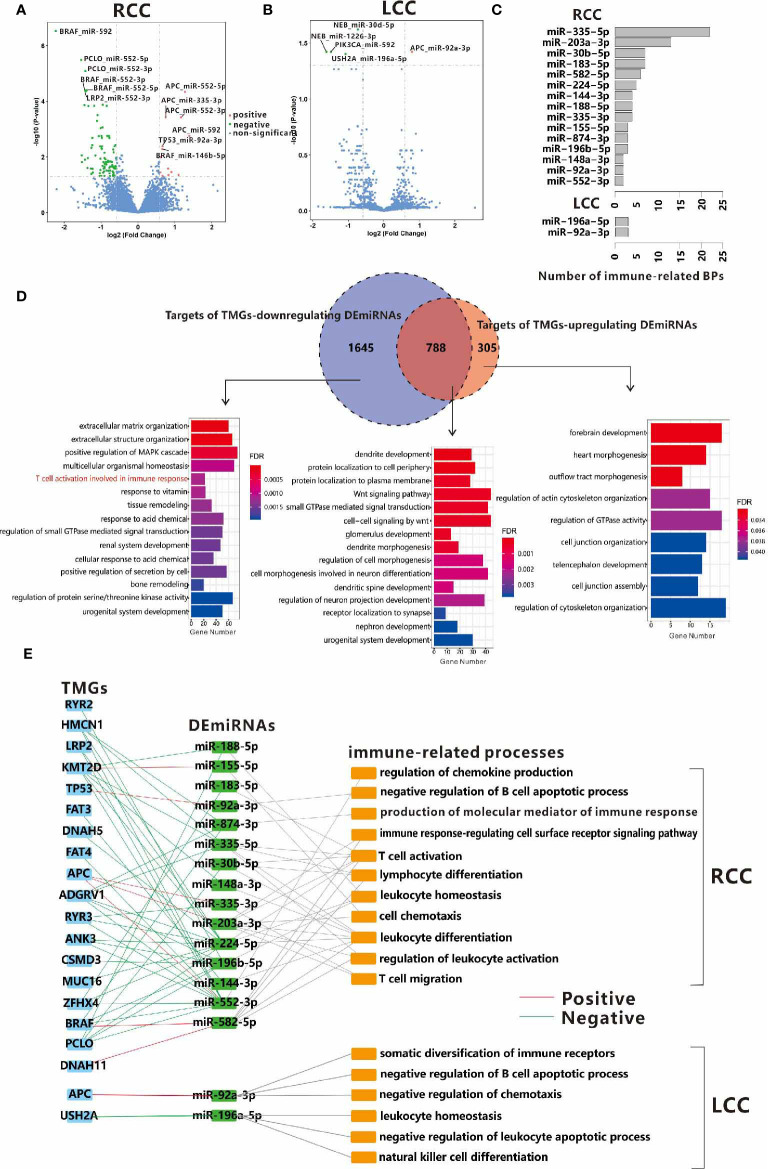
Associations between TMGs and DEmiRNAs in RCC **(A)** and LCC **(B)**. **(C)** The number of immune-related BPs in target genes of DEmiRNAs regulated by TMGs in RCC and LCC. **(D)** Venn diagrams show the overlap between the targets of TMG-downregulating DEmiRNAs and the targets of TMG-upregulating DEmiRNAs in RCC. The bar plots show the GO functional enrichment analysis of the corresponding parts of genes in the Venn diagram and the immune-related BPs are marked in red. **(E)** The crosslinks among TMGs, DEmiRNAs, and immune-related BPs in RCC and LCC. The red line represents positive relationships while the green line represents negative relationships.

The regulatory network in biology is exceedingly complex as any alterations in gene expression could be initiated or influenced by multiple regulators such as transcription factors, miRNAs, etc. MiRNA is a kind of ncRNA that modulates the expression levels of up to 60% of the protein-coding genes at both the transcription and post-transcription level (31, 32). So, the associations of TMG-DEG relationships identified above may be indirect and connected through miRNAs. Based on small RNA-seq data, we first investigated the DEmiRNAs in LCC and RCC by comparing their expressions level in cancer tissues to those in the normal control tissues. We found that 209 and 167 miRNAs were up-regulated and down-regulated in RCC, while only 119 up-regulated and 121 down-regulated miRNAs were found in LCC. Next, we identified the TMG-DEmiRNA relationships in LCC and RCC using a similar method which was used to identify TMG-DEG pairs. As expected, the number of significant TMG-DEmiRNA pairs in RCC was much more than that in LCC (109 vs. 5), further suggesting the tumorigenesis mechanism in RCC may be more complex. In RCC, 13 positive and 96 negative TMG-DEmiRNA associations were obtained (**Figure 5A** and **Supplementary Table 6**), of which 4 DEmiRNAs were common. While in LCC, only 1 positive and 4 negative TMG-DEmiRNA pairs were found (**Figure 5B** and **Supplementary Table 6**). Among them, miR-552-5p was associated with the most TMGs in RCC. Although no study has reported on the function of miR-552-5p in CC until now, its strong relationship with cancerous pathways such as cell proliferation and metastasis and its potentiality in prognostic prediction have been widely acknowledged (33, 34), suggesting that miR-552-5p likely plays an important role in CC.

To further understand the roles of TMGs regulating DEmiRNAs, we attempted to identify the potential targets of these DEmiRNAs. Considering that most known miRNA-target pairs were experimentally verified in cancer-related processes, there may be some biases if we included all the known targets of DEmiRNAs. Therefore, we only considered the miRNA-target relationships that were predicted by miRANDA (35) or recorded in miRDB in this study (36). To improve prediction accuracy, we also required that the DEmiRNA and its target within the same pair should be differentially expressed in the opposite direction in each cohort (If DEmiRNAs were up-regulated, target genes were down-regulated, vice versa). Finally, 1475 and 4836 miRNA-target relationships were obtained for the up-regulated and down-regulated DEmiRNAs of TMGs mutation in RCC respectively, among them, 1093 and 2433 target genes were involved respectively (**Supplementary Table 7**). However, only 854 miRNA-target relationships with 741 genes were found in LCC (**Supplementary Table 7**), of which 148 and 641 are targets of up-regulated and down-regulated DEmiRNAs of TMGs mutation. In RCC, 788 genes were the common targets of both TMG-upregulating and TMG-downregulating DEmiRNAs. Functional enrichment analysis showed that the enriched terms were Wnt signaling pathway and some neuron-related biological processes (**Figure 5D** middle), suggesting that these pathways may be important in tumorigenesis and are regulated by multiple factors. Specifically, we found the unique targets of TMG-downregulating DEmiRNAs in RCC were involved in immune response (**Figure 5D** left). However, no immune-related biological process was over-represented in the targets of DEmiRNAs in LCC (**Figure S7**), suggesting that some immune-related genes may be up-regulated through the down-regulation of DEmiRNAs by TMGs mutations in RCC.

To determine which mutation of TMG affects TIME through the regulation of miRNAs, we also performed a functional enrichment analysis on the targets of each DEmiRNA. We found the following, 15 out of 38 TMG-associated DEmiRNAs (39.5%) in RCC and only 2 TMG-associated DEmiRNAs (40%) in LCC were related to at least one immune-related process (**Supplementary Table 8** and **Figure 5C**). In detail, 6 immune-related DEmiRNAs were up-regulated in the mutation groups of 5 TMGs including *APC*, *TP53*, *DNAH11*, *BRAF*, and *KMT2D*, while down-regulation of 11 immune-related DEmiRNAs were associated with mutations of 15 TMGs in RCC (**Figure 5E**). The major immune-related processes of these DEmiRNAs were T cell activation, T cell migration, leukocyte activation, etc. (**Figure 5E**). As an example, miR-183-5p was annotated with T cell activation, T-helper 17 type immune response, T-helper 17 cell differentiation, etc. Existing reports have also confirmed its role in the regulation of Th17 differentiation (37). In this study, we found the mutation of *ZFHX4* promoted down-regulation of miR-183-5p in RCC. Additionally, miR-148a-3p has been reported to regulate *PD-L1* in CC (38) and functions as an important immune regulator (39). We also found that the mutation of *PCLO* was associated with low expression of miR-148a-3p. However, only two associations (*APC* and miR-92A-3p, *USH2A* and miR-196a-5p) were found in LCC (**Figure 5E**). Our results showed a larger crosslink between genetic alternation and miRNA expression aberrance for TIME regulation in RCC.

### TMGs Influence TIME Through Regulation of DNA Methylation

**Figure 6:**
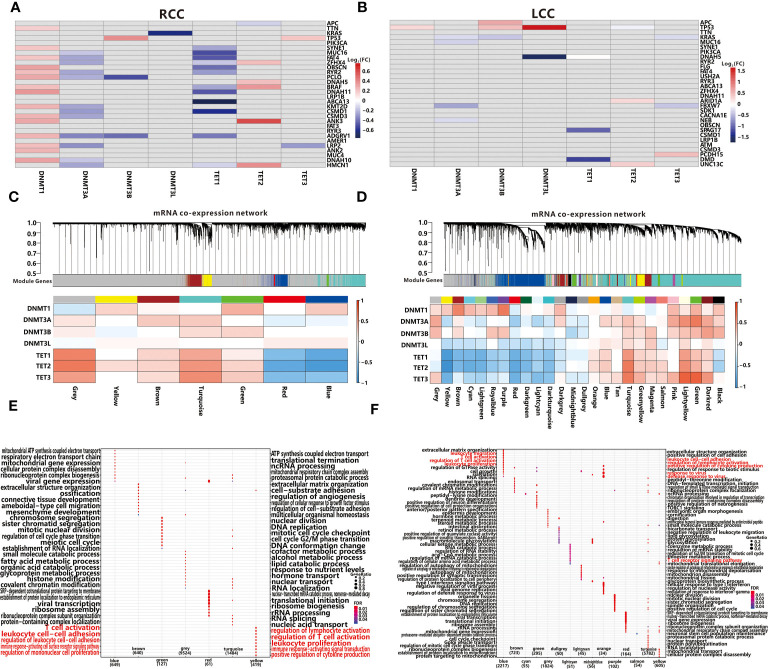
Associations between TMGs and 7 methylation-associated genes using the Wilcoxon test in RCC **(A)** and LCC **(B)**. The grey cells represent insignificant associations while the colored cells represent significant associations with p<0.05. The red color represents a higher expression in mutation group while the blue color represents a lower expression. WGCNA resolves co-expressed modules in RCC **(C)** and LCC **(D)**. Top panels describe modules defined by dynamic tree cutting and the grey color represents genes not belonging to any modules. Bottom heatmaps represent associations between modules and 7 methylation-associated genes. Cells with black edge represent significant associations with *p*<0.05. GO functional enrichment analysis of each module in RCC **(E)** and LCC **(F)**.

DNA methylation is a major type of epigenetic modification and plays a crucial role in tumorigenesis (40). According to the previous reports (41), DNA methyltransferases (DNMT family) and demethylases (TET family) often mediate DNA methylation abnormalities through co-expression with other genes. To explore the potential mechanism of DNA methylation changes, we further analyzed whether DNMT family (*DNMT1, DNMT3A, DNMT3B, DNMT3L*) or TET family (*TET1, TET2, TET3*) expression were affected by TMG mutations. In RCC, expression of *DNMT1* and *TET2* were activated by mutations of 15 and 5 TMGs respectively, while expression of *DNMT3A, DNMT3L* and *TET1* were inhibited by mutations of 1, 12, 12 TMGs respectively (**Figure 6A**). However, the associations between methylation-associated genes and TMGs were weaker in LCC than those in RCC (**Figure 6B**). Next, we conducted weighted gene co-expression network analyses (WGCNA) based on the expression datasets of LCC and RCC by “WGCNA” R package. In RCC, a total of 6 modules from 3380 genes were identified in the co-expression network, while 9524 genes were not assigned to any modules (presented as grey color). As shown in **Figure 6C**, all modules were significantly associated with the expression level of *TET2*, whereas no module was correlated to *DNMT3L*. Furthermore, we found that TET family had a stronger correlation with most modules than DNMT family. Functional enrichment analysis showed that immune related BPs were associated with yellow module (**Figure 6E**), and the module was positively correlated with *DNMT1* and *TET2*, which were also up-regulated by TMGs. Therefore, we hypothesized that expression levels of the two methylation-associated genes may be involved in immune-related processes. In LCC, a total of 25 modules from 12909 genes were identified in the co-expression network, while 1824 genes were not assigned to any modules (presented as grey color, **Figure 6D**). Based on functional enrichment analysis, we found that immune related BPs were related to blue module (**Figure 6F**), and the module was positively correlated with *DNMT3A* and TET family. Notably, *TET2* was positively correlated with immune-related module in both RCC and LCC, which may play an important role in TIME.

**Figure 7:**
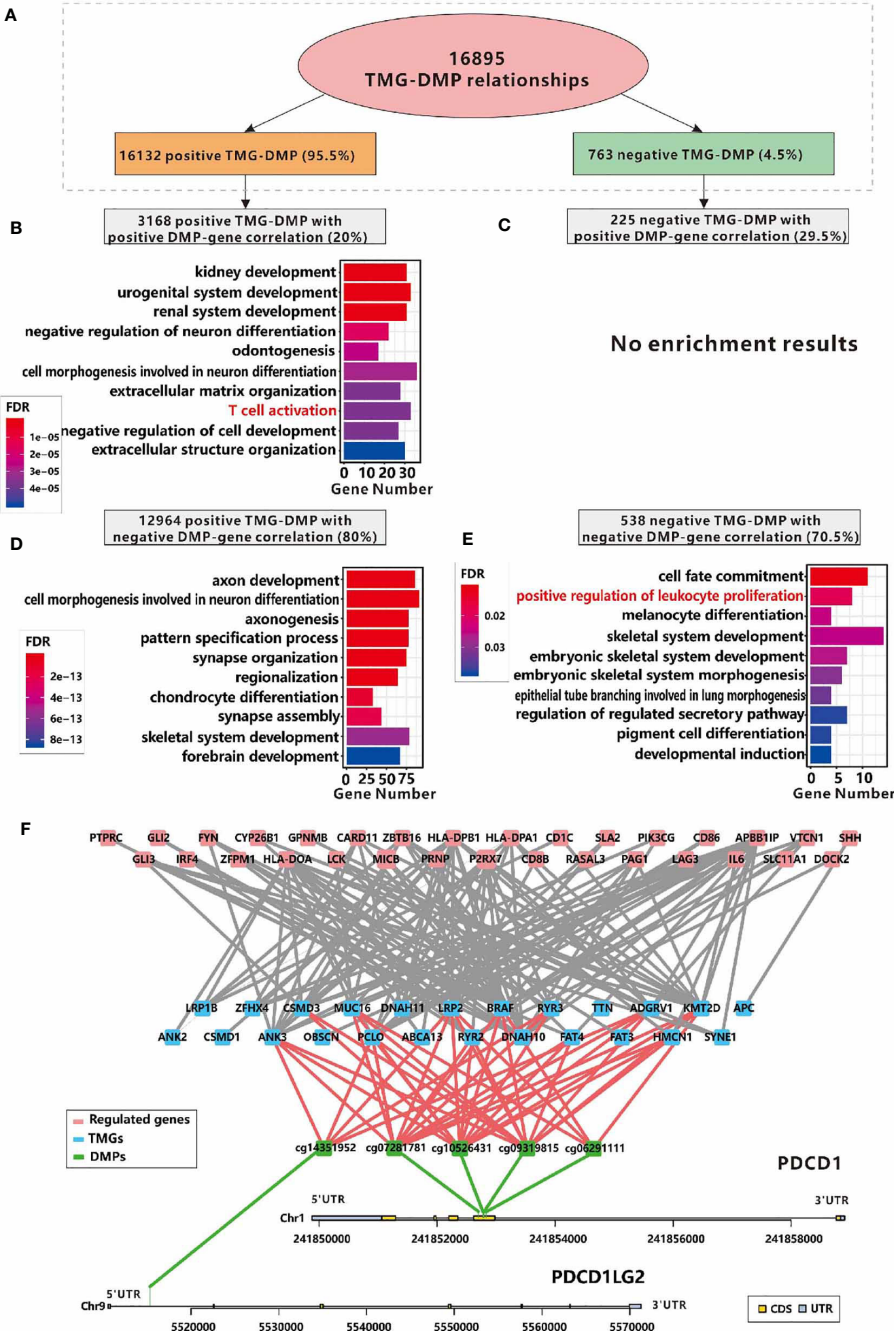
Relationships between immune infiltration and DMPs regulated by TMGs in RCC. **(A)** The number and the proportions of positive and negative TMG-DMP relationships. **(B)** The GO functional enrichment analysis of the genes which are positively correlated with the DNA methylation level of TMG-upregulating DMPs. **(C)** No significant result was found in the genes which are positively correlated with the DNA methylation level of TMG-downregulating DMPs. **(D)** The GO functional enrichment analysis of the genes which are negatively correlated with the DNA methylation level of TMG-upregulating DMPs. **(E)** The GO functional enrichment analysis of the genes which are negatively correlated with the DNA methylation level of TMG-downregulating DMPs. **(F)** The network among TMGs (blue), DNA methylation probes (green) and genes (red) whose functions are annotated with T cell activation, of these, DNA methylation probes are derived from *PDCD1* and *PDCD1LG2*.

Excepting for affecting methylation related genes, the mutations of specific genes may trigger DNA methylation alternation directly (42). After performing an analysis of the DNA methylation profiles in LCC and RCC from TCGA portal, we initially obtained 177996 and 137239 DMPs in RCC and LCC respectively. Next, 5506 and 52858 TMG-DMP pairs were identified in LCC and RCC with FDR<0.05 and △ß value >0.15 respectively. The same as before, the number of TMG-DMP associations identified in RCC was much higher compared to LCC. Considering that DNA methylation disruption usually causes an aberrant expression of the corresponding gene, we calculated the PCC between the methylation level of DMP and the expression level of its corresponding gene in each cohort to further filter the TMG-DMP associations. 911 and 16895 TMG-DMP pairs in LCC and RCC respectively were remained, with the threshold of an adjusted *P*-value less than 0.05 (**Supplementary Table 9**). Interestingly, up to 95.5% in RCC and 71.1% in LCC of TMG-DMP associations were positive (**Figure 7A** and **Figure S8A**).

When taking the PCC of DNA methylation signals and the corresponding gene expression levels into account, we found that the DMPs with positive associations with both TMG mutations and corresponding gene expression, and the DMPs with negative association with both TMG mutations and corresponding gene expression were related to immune-related processes such as T cell activation, positive regulation of leukocyte proliferation in RCC (**Figures 7B, E**). However, no immune-related processes were over-represented in other groups of TMG-DMP associations in RCC (**Figures 7C, D**) and any group in LCC (**Figure S8**). This suggested that TMG mutation can also up-regulate immune-related genes *via* the disruption of the corresponding DNA methylation level in RCC. **Figure 7F** shows the sub-network of ‘T cell activation’ formed by positive TMG-DMP associations with DMP also positively correlated with gene expression in RCC. Several known immune-related genes, especially the immune therapy checkpoint *PDCD1* (also called *PD-1*) and its ligand *PDCD1LG2* (also called *PD-L2*) were involved. Our results showed that 4 probes located in the body region of *PD-1* (cg10526431, cg09319815, cg07281781, and cg06291111) were up-regulated by 12 TMGs, while 1 probe (cg14351952) located in the 5’UTR of *PD-L2* was up-regulated by 5 TMGs, which in turn led to an elevated expression of *PD-1* and *PD-L2* in RCC (**Figure 7F**). Furthermore, another well-known immune checkpoint molecular *LAG3* was also found to be up-regulated by mutation of *PCLO* through the disruption of the DNA methylation level of cg14292870 in RCC (**Figure 7F**).

**Figure S9** shows the sub-network of ‘positive regulation of leukocyte proliferation’ caused by TMG-DMP negative associations with DMP inverse correlation with gene expression in RCC. A total of 7 TMGs and 8 immune-related genes were involved. Interestingly, we found several distinct methylation probes in *HLA-DPA1* were both regulated by TMG mutation positively and negatively, where cg22941409 was positively regulated by mutations of *ADGRV1*, *ANK3*, *DNAH10*, *DNAH11*, *LRP1B*, *LRP2* and *KMT2D*, and cg01804934 was inversely regulated by mutation of *BRAF* (**Figure S10A**). However, the methylation level of cg22941409 was positively correlated with the expression level of *HLA-DPA1* (**Figure S10B**) but cg01804934 was reversed (**Figure S10C**), both resulted in the elevated expression of *HLA-DPA1* in RCC. Furthermore, we also found 2 probes located in *HLA-DPB1* that were down-regulated by mutations of *BRAF* and *ANK3* while 6 probes were up-regulated by mutations of 13 TMGs (**Figure S11A**), all of which also resulted in a higher expression of *HLA-DPB1* in RCC (**Figures S11B, C**). These results showed that the crosslink between DNA methylation and gene mutation is important for TIME in RCC.

### Integrative Analysis of Immune-Related TMG-Centric Regulatory Network

**Figure 8:**
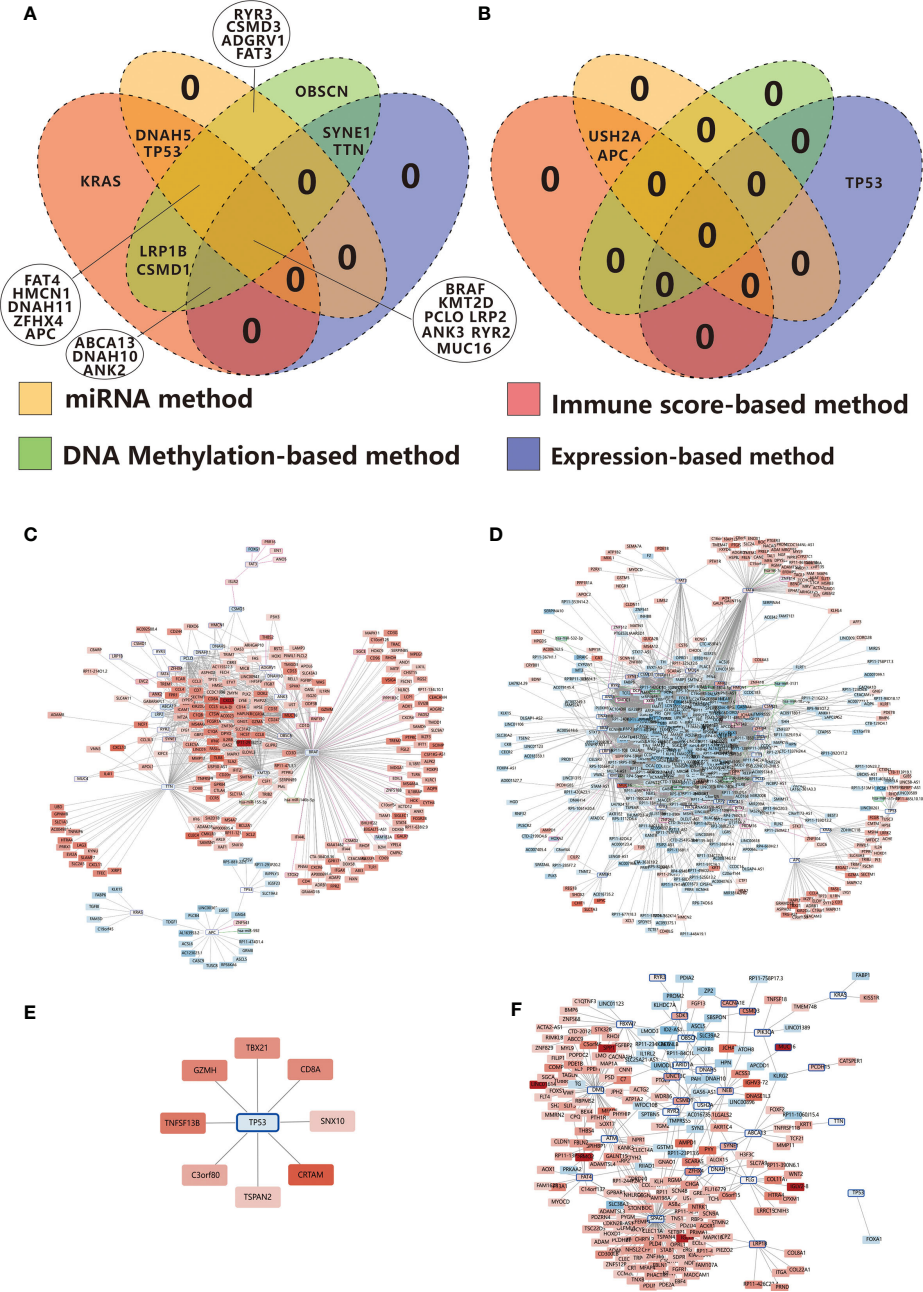
Venn diagrams show the overlapping immune-related TMGs identified using the immune score-based method, gene expression-based method, miRNA target-based method, and DNA methylation-based method in RCC **(A)** and LCC **(B)**. **(C)** Positive correlation network centered on immune-related TMGs in RCC. **(D)** Negative correlation network centered on immune-related TMGs in RCC. **(E)** Positive correlation network centered on immune-related TMGs in LCC. **(F)** Negative correlation network centered on immune-related TMGs in LCC. The color strength represents the fold change of expression in the high-immunity group compared to that in the low-immunity group. Red indicates up-regulation while blue suggests down-regulation in the high-immunity group. The nodes with a blue edge represent TMGs, the nodes with no edge represent DEGs, the nodes with a purple edge represent the genes regulated by TMG-regulating DMPs while those with green edges represent miRNAs.

According to the results above, we can make a conclusion that the mutations of some TMGs in RCC are associated with immune-related genes directly or indirectly through miRNA and DNA methylation. We found 20, 12, 18, 24 immune-related TMGs in the TMG-IS correlation analysis, TMG-DEG correlation analysis, TMG-DEmiRNA correlation analysis, and TMG-DMP correlation analysis respectively. Among them, 7 TMGs (*BRAF, PCLO, MUC16, LRP2, ANK3, KMT2D, RYR2*) were common in the four kinds of analysis (**Figure 8A**). While in LCC, only 3 common immune-related TMGs were observed (*APC, TP53, USH2A*, **Figure 8B**).

In order to get a greater insight into the regulatory mechanism of TMGs on TIME, we constructed an immune-related TMG-centric regulatory network in RCC and LCC respectively. In RCC, the immune-related TMG-centric regulatory network consists of 4289 TMG-DEG, 44 TMG-DEmiRNA, and 213 TMG-DMP (70 TMG-corresponding genes) relationships. As shown in **Figure 8**, most targets in the positive TMG-DEG relationships were associated with high expression levels in the high-immunity group (**Figure 8C**), while the genes within negative TMG-DEG relationships were associated with low expression levels in the high-immunity group (**Figure 8D**). In contrast, *APC*, *TP53*, and *KRAS* exhibited opposite relationships, further verifying that the three genes play a negative role on TIME in RCC (**Figures 8C, D**). However, in LCC, only 327 TMG-DEG relationships and 1 TMG-DMP relationship were identified (**Figures 8E, F**). Of note, while *TP53* exhibited a negative correlation with immunity in RCC, it seemed to play a positive role on TIME in LCC as the targets in the positive relationships were up-regulated in the high-immunity group (**Figure 8E**) while those involved in the negative relationships were down-regulated in the high-immunity group (**Figure 8F**). The conclusion runs contrary to previous findings that TP53 mutation suppresses immunity in colon cancer (13).

### Effects of APC, BRAF and TP53 Mutations on Immunotherapy Efficacy in CC

The analyses of TMG regulatory network based on multi-omics data above suggest some TMGs such as *APC* have suppressive effects on cancer immunity while some TMGs such as *BARF* play opposite roles. It led us to hypothesize that TMG mutations may be served as predictive biomarkers for immunotherapy efficacy in CC. To directly address this point and validate our hypothesis, we collected the survival data of CC patients who received non-immunotherapy or immunotherapy, with mutations of some TMGs known, from cBioPortal database. Then we investigated the impacts of TMG mutations on the efficacy of immunotherapy through Kaplan-Meier analyses.

**Figure 9:**
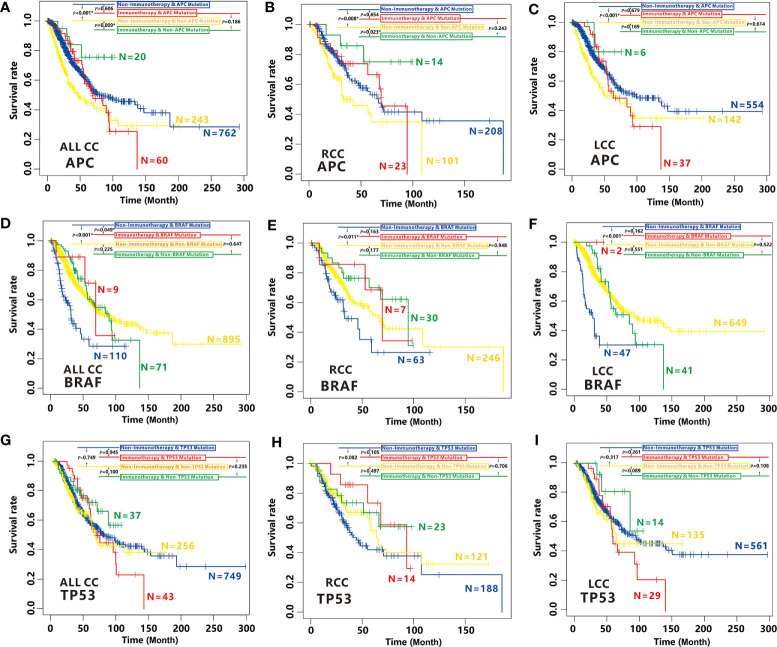
Effects of *APC*, *BRAF* and *TP53* mutation on immunotherapy efficacy in CC. **(A–C)** Comparison of survival curves among *APC*-mutated patients with non-immunotherapy (blue), *APC*-mutated patients with immunotherapy (red), non-*APC*-mutated patients with non-immunotherapy (yellow) and, non-*APC*-mutated patients with immunotherapy (green) by Kaplan–Meier analysis in CC cohort **(A)**, RCC cohort **(B)** and LCC cohort **(C)**. **(D–F)** Comparison of survival curves among *BRAF*-mutated patients with non-immunotherapy (blue), *BRAF*-mutated patients with immunotherapy (red), non-*BRAF*-mutated patients with non-immunotherapy (yellow) and, non-*BRAF*-mutated patients with immunotherapy (green) by Kaplan–Meier analysis in CC cohort **(D)**, RCC cohort **(E)** and LCC cohort **(F)**. **(G–I)** Comparison of survival curves among *TP53*-mutated patients with non-immunotherapy (blue), *TP53*-mutated patients with immunotherapy (red), non-*TP53*-mutated patients with non-immunotherapy (yellow) and, non-*TP53*-mutated patients with immunotherapy (green) by Kaplan–Meier analysis in CC cohort **(G)**, RCC cohort **(H)** and LCC cohort **(I)**.

As expected, we found immunotherapy can effectively improve the overall survival (OS) for non-*APC*-mutated patients (P=0.009, **Figure 9A**) but not for *APC*-mutated patients in CC (P=0.606, **Figure 9A**). On the contrary, for CC patients with non-immunotherapy treatment, *APC* mutation is a favorable prognostic factor (P<0.001, **Figure 9A**). All the findings were also established in RCC cohort (**Figure 9B**). A slight improvement of OS for non-*APC*-mutated patients with immunotherapy was also observed compared to *APC*-mutated patients who treated with the same immunotherapy in LCC, although not significant due to the small sample size (**Figure 9C**), suggesting non-*APC* mutation can be considered as a biomarker for immunotherapy efficacy in both LCC and RCC.

In contrary to *APC, BRAF* mutation is associated with high cancer immunity. Not surprisingly, we found *BRAF*-mutated patients with immunotherapy have higher OS time than those with non-immunotherapy (P=0.049, **Figure 9D**). While the OS is not better for non *BRAF-*mutated patients with immunotherapy than those with non-immunotherapy (P=0.225, **Figure 9D**). Unfortunately, we didn’t find significant differences between the OS of the *BRAF*-mutated patients with immunotherapy and those with non-immunotherapy in RCC or LCC cohort, it is most likely because of small sample size of *BRAF*-mutated patients with immunotherapy. However, the similar tendencies were also observed (**Figures 9E, F**), indicating *BRAF* mutation is also a biomarker of immunotherapy efficacy for CC patients.

However, mutation of *TP53* had no effect on the efficacy of immunotherapy in all CC patients, RCC or LCC patients (**Figures 9G–I**). Therefore, *TP53* cannot be considered as a biomarker for immunotherapy efficacy in CC.

## Discussion

Tremendous variations in the microbiome, clinical factors, microenvironment, genomic characteristics and molecular events have been reported between LCC and RCC in the last decade (43). Among them, molecular event, which includes multiple kinds of alternations such as DNA variation, gene expression, and DNA methylation, is the most important mechanism underlying between LCC and RCC. However, the relationships among different types of molecular events, and their impact on TIME in LCC and RCC has not been systematically studied. In this study, we revealed the distinct differences in the immune cell infiltration levels especially CD8+ T cells between RCC and LCC. Then, we classified the 30 most frequently mutated genes as TMGs in each cohort, and analyzed the relationship between TMG mutation and the alternations in the gene expression, miRNA regulation, and DNA methylation. Our results showed that the mutations of TMGs in RCC can regulate more genes and affect more pathways compared to LCC, which was consistent with a previous study that demonstrating RCC had a more diverse mechanism in tumorigenesis (7). Additionally, we also showed that TMG mutation was strongly associated with TIME in both cohorts, and especially in RCC. Based on the above relationships, we further validated the conclusion that non-*APC* mutation and *BRAF* mutation can serve as biomarkers for immunotherapy but not *TP53*.

Until now, a growing body of evidence suggests that gene mutation is strongly associated with TIME (44). In a previous study on lung adenocarcinoma, several commonly mutated genes were predicted to be neoantigens of the major histocompatibility complex (MHC) Class I and Class II molecules (45), and some of these genes are also immune-related TMGs in RCC, including *MUC16*, *ZFHX4*, *FAT3*, *TTN*, and *RYR2*. Specifically, *RYR2* and *RYR3*, the members of ryanodine receptors (RyR), have been reported to be associated with T cell activation (46). In this study, we found that mutations of most TMGs were associated with a higher expression of immune-related genes, among which 7 immune-related TMGs consisted of *BRAF*, *PCLO*, *MUC16*, *LRP2*, *ANK3*, *KMT2D*, *RYR2* were observed in multiple methods. *BRAF* was a well-known regulator in CC and its mutation has been considered to be a reliable biomarker for prognosis and therapy (47). Regarding *ANK3*, although it is currently not well studied, existing studies have suggested that it is a potential therapeutic target oncogene in CC (48). However, the genes such as *APC*, *TP53*, and *KRAS* exhibit a negative effect on TIME in RCC. Interestingly, our study showed that *TP53* may promote tumor immunity in LCC. Mutation of *TP53* was associated with a higher expression of 11 genes, among which, 8 genes were up-regulated in the high-immunity group of LCC and were enriched in the gene set of T cell activation and leukocyte differentiation. Meanwhile, the unique down-regulated gene in the group of *TP53* mutation in LCC, *FOXA1*, which has been reported to inhibit T cells proliferation in lung cancer (49), was also observed to be down-regulated in the high-immunity group of LCC (**Figure 8F**), indicating that *TP53* mutation may exhibit a suppressive function against immunity inhibition in LCC.

Acting as a key regulator at the post-transcriptional level, miRNAs participate in many biological processes including the immune system, and play a major role in the cancer-immune response (50). We found 15 immune-related miRNAs that were associated with TMGs mutations in RCC. Among them, *TP53* and *APC* could only up-regulate immune-related miRNAs, suggesting that they may be able to down-regulate immune-related genes through miRNAs (**Figure 5E**). However, the genes such as *KMT2D*, *DNAH11*, and *BRAF*, which have a positive correlation with tumor immunity level, also up-regulated several immune-related miRNAs (**Figure 5E**). They may function together with other miRNAs by forming negative feedback loops to regulate immune-related genes.

DNA methylation is one of the universal epigenetic types which can regulate gene expression and genomic stability. CIMP, one of the CC molecular subtypes, is found to be correlated with *BRAF* mutation and MSI phenotype in the early years (51), suggesting that there is a relationship between gene mutation and the disruption of DNA methylation in CC. Remarkably, we found that the DNA methylation levels of the immune molecular checkpoints such as *PD-1* and *LAG3* were regulated by mutations of some TMGs in RCC, which in turn influenced the expression of their corresponding genes and resulted in higher immunogenicity. Therefore, mutations of these TMGs may act as biomarkers of immunotherapy and immune cell infiltration.

The identified studies indicated that *POLE* and *POLD1* mutations are biomarkers for immunotherapy across multiple cancer types (52), and *BRAF* mutation can improve antitumor activity of immunotherapy in melanoma (15). In our study, we found that *APC* mutation *exerted* a suppressive effect on immunotherapy while the role of *BRAF* mutation on immunotherapy *are* inversely. Taken together, we propose immunotherapy benefits for CC patients with non-*APC* mutation or *BRAF* mutation. However, *TP53* mutation had no effect on the efficacy of immunotherapy.

## Conclusions

In conclusion, we systematically compared the associations between the mutational profiles and tumor immunity based on the multi-omics data in LCC and RCC respectively and found several distinct regulatory mechanisms underlying the TIME in both types of CC. Our results will provide valuable clues that would be helpful for the identification of novel targets for immunotherapy.

## Data Availability Statement

The datasets presented in this study can be found in online repositories. The names of the repository/repositories and accession number(s) can be found in the article/**Supplementary Material**.

## Ethics Statement

The studies involving human participants were reviewed and approved by School of Medicine, Ningbo University. The patients/participants provided their written informed consent to participate in this study.

## Author Contributions

QL and TY wrote the manuscript. YZ, YL, SH, and HR did the bioinformatics analysis. QL, XD, and GZ designed the idea of this study. DN improved English writing in manuscript. JL, XF, YYX, YX, JM, LeC, CC, SN provided some suggestions for the study. MY and LvC contributed to the revised manuscript and provided the corresponding data analysis. All authors contributed to the article and approved the submitted version.

## Funding

This work was supported by the Fundamental Research Funds for the Provincial Universities of Zhejiang [grant number: SJLZ2021001], the National Natural Science Foundation of China [grant number: 31970630], the Zhejiang Provincial Natural Science Foundation of China [grant number: LY21C060002, LY19H160011], the Ningbo Health Branding Subject Fund [grant number: PPXK2018-05], the Zhejiang Key Laboratory of Pathophysiology [grant number: 201812], the Ningbo Clinical Research Center for Digestive System Tumors [grant number: 2019A21003], the Key Laboratory of Diagnosis and Treatment of Digestive System Tumors of Zhejiang Province [grant number: Grant No. 2019E10020], the Graduate Research Innovation Fund of Ningbo University [grant number: IF2020163] and K. C. Wong Magna Fund in Ningbo University.

## Conflict of Interest

The authors declare that the research was conducted in the absence of any commercial or financial relationships that could be construed as a potential conflict of interest.

## Abbreviations

LCC, left-sided colon cancer; RCC, right-sided colon cancer; TIME, Tumor immune microenvironment; CC, Colon cancer; TMB, tumor mutation burden; TMGs, top mutated genes; TCGA, The Cancer Genome Atlas; *MAF*, Mutation Annotation Format; MSI, Microsatellite Instability; CIN, chromosomal instability; MSI-H, microsatellite instability-high; MSI-L, microsatellite instability-low; MSS, microsatellite stability; CIMP, CpG island methylator phenotype; CIMP-H, high level of CIMP; CIMP-L, low level of CIMP; FDR, false discovery rate; SMF, somatic mutation frequency; Mbp, megabase pairs; FC, Fold Change; DEGs, differentially expressed genes; DEmiRNAs, differentially expressed miRNAs; GO, Gene Ontology; BP, Biological Process; DMRs, differentially methylated regions; DMPs, differentially methylated probes; MHC, major histocompatibility complex; RyR, ryanodine receptors.

## References

[1] Siegel RL Miller KD Jemal A. Cancer Statistics, 2019. CA: Cancer J Clin (2019) 69(1):7–34. 10.3322/caac.21551 30620402

[2] Zhou M Hu L Zhang Z Wu N Sun J Su J. Recurrence-Associated Long non-Coding RNA Signature for Determining the Risk of Recurrence in Patients With Colon Cancer. Mol Ther Nucleic Acids (2018) 12:518–29. 10.1016/j.omtn.2018.06.007 PMC6076224 30195788

[3] Jacobs ET Thompson PA Martinez ME. Diet, Gender, and Colorectal Neoplasia. J Clin Gastroenterol (2007) 41(8):731–46. 10.1097/MCG.0b013e3180338e56 17700421

[4] Dong L Jin X Wang W Ye Q Li W Shi S. Distinct Clinical Phenotype and Genetic Testing Strategy for Lynch Syndrome in China Based on a Large Colorectal Cancer Cohort. Int J Cancer (2020) 146(11):3077–86. 10.1002/ijc.32914 32030746

[5] Gupta S Balasubramanian BA Fu T Genta RM Rockey DC Lash R. Polyps With Advanced Neoplasia Are Smaller in the Right Than in the Left Colon: Implications for Colorectal Cancer Screening. Clin Gastroenterol Hepatol Off Clin Pract J Am Gastroenterol Assoc (2012) 10(12):1395–401.e2. 10.1016/j.cgh.2012.07.004 PMC3595198 22835574

[6] Benedix F Kube R Meyer F Schmidt U Gastinger I Lippert H. Comparison of 17,641 Patients With Right- and Left-Sided Colon Cancer: Differences in Epidemiology, Perioperative Course, Histology, and Survival. Dis Colon Rectum (2010) 53(1):57–64. 10.1007/DCR.0b013e3181c703a4 20010352

[7] Hu W Yang Y Li X Huang M Xu F Ge W. Multi-Omics Approach Reveals Distinct Differences in Left- and Right-Sided Colon Cancer. Mol Cancer Res MCR (2018) 16(3):476–85. 10.1158/1541-7786.MCR-17-0483 29187560

[8] Mukund K Syulyukina N Ramamoorthy S Subramaniam S. Right and Left-Sided Colon Cancers - Specificity of Molecular Mechanisms in Tumorigenesis and Progression. BMC Cancer (2020) 20(1):317. 10.1186/s12885-020-06784-7 32293332 PMC7161305

[9] Fridman WH Zitvogel L Sautes-Fridman C Kroemer G. The Immune Contexture in Cancer Prognosis and Treatment. Nat Rev Clin Oncol (2017) 14(12):717–34. 10.1038/nrclinonc.2017.101 28741618

[10] Zhang Z Bao S Yan C Hou P Zhou M Sun J. Computational Principles and Practice for Decoding Immune Contexture in the Tumor Microenvironment. Briefings Bioinf (2020). 10.1093/bib/bbaa075 32496512

[11] Liang L Zeng JH Qin XG Chen JQ Luo DZ Chen G. Distinguishable Prognostic Signatures of Left- and Right-Sided Colon Cancer: A Study Based on Sequencing Data. Cell Physiol Biochem Int J Exp Cell Physiol Biochem Pharmacol (2018) 48(2):475–90. 10.1159/000491778 30016783

[12] Zhang L Zhao Y Dai Y Cheng JN Gong Z Feng Y. Immune Landscape of Colorectal Cancer Tumor Microenvironment From Different Primary Tumor Location. Front Immunol (2018) 9:1578. 10.3389/fimmu.2018.01578 30042763 PMC6048410

[13] Li L Li M Wang X. Cancer Type-Dependent Correlations Between Tp53 Mutations and Antitumor Immunity. DNA Repair (2020) 88:102785. 10.1016/j.dnarep.2020.102785 32007736

[14] Picard E Verschoor CP Ma GW Pawelec G. Relationships Between Immune Landscapes, Genetic Subtypes and Responses to Immunotherapy in Colorectal Cancer. Front Immunol (2020) 11:369. 10.3389/fimmu.2020.00369 32210966 PMC7068608

[15] Ribas A Lawrence D Atkinson V Agarwal S Miller WH Jr Carlino MS. Combined BRAF and MEK Inhibition With PD-1 Blockade Immunotherapy in BRAF-mutant Melanoma. Nat Med (2019) 25(6):936–40. 10.1038/s41591-019-0476-5 PMC8562134 31171879

[16] Zhou M Zhang Z Bao S Hou P Yan C Su J. Computational Recognition of Lncrna Signature of Tumor-Infiltrating B Lymphocytes With Potential Implications in Prognosis and Immunotherapy of Bladder Cancer. Briefings Bioinf (2020). 10.1093/bib/bbaa047 32382761

[17] Sun J Zhang Z Bao S Yan C Hou P Wu N. Identification of Tumor Immune Infiltration-Associated lncRNAs for Improving Prognosis and Immunotherapy Response of Patients With Non-Small Cell Lung Cancer. J Immunother Cancer (2020) 8(1):e000110. 10.1136/jitc-2019-000110 32041817 PMC7057423

[18] Weisenberger DJ Siegmund KD Campan M Young J Long TI Faasse MA. Cpg Island Methylator Phenotype Underlies Sporadic Microsatellite Instability and Is Tightly Associated With Braf Mutation in Colorectal Cancer. Nat Genet (2006) 38(7):787–93. 10.1038/ng1834 16804544

[19] Yoshihara K Shahmoradgoli M Martinez E Vegesna R Kim H Torres-Garcia W. Inferring Tumour Purity and Stromal and Immune Cell Admixture From Expression Data. Nat Commun (2013) 4:2612. 10.1038/ncomms3612 24113773 PMC3826632

[20] Newman AM Steen CB Liu CL Gentles AJ Chaudhuri AA Scherer F. Determining Cell Type Abundance and Expression From Bulk Tissues With Digital Cytometry. Nat Biotechnol (2019) 37(7):773–82. 10.1038/s41587-019-0114-2 PMC6610714 31061481

[21] Miao YR Zhang Q Lei Q Luo M Xie GY Wang H. Immucellai: A Unique Method for Comprehensive T-Cell Subsets Abundance Prediction and Its Application in Cancer Immunotherapy. Adv Sci (2020) 7(7):1902880. 10.1002/advs.201902880 PMC7141005 32274301

[22] Mayakonda A Lin DC Assenov Y Plass C Koeffler HP. Maftools: Efficient and Comprehensive Analysis of Somatic Variants in Cancer. Genome Res (2018) 28(11):1747–56. 10.1101/gr.239244.118 PMC6211645 30341162

[23] Morris TJ Butcher LM Feber A Teschendorff AE Chakravarthy AR Wojdacz TK. ChAMP: 450k Chip Analysis Methylation Pipeline. Bioinformatics (2014) 30(3):428–30. 10.1093/bioinformatics/btt684 PMC3904520 24336642

[24] Dedeurwaerder S Defrance M Calonne E Denis H Sotiriou C Fuks F. Evaluation of the Infinium Methylation 450k Technology. Epigenomics (2011) 3(6):771–84. 10.2217/epi.11.105 22126295

[25] Meguid RA Slidell MB Wolfgang CL Chang DC Ahuja N. Is There a Difference in Survival Between Right- Versus Left-Sided Colon Cancers? Ann Surg Oncol (2008) 15(9):2388–94. 10.1245/s10434-008-0015-y PMC3072702 18622647

[26] Wielandt AM Hurtado C Moreno CM Villarroel C Castro M Estay M. Characterization of Chilean Patients With Sporadic Colorectal Cancer According to the Three Main Carcinogenic Pathways: Microsatellite Instability, CpG Island Methylator Phenotype and Chromosomal Instability. Tumour Biol J Int Soc Onco dev Biol Med (2020) 42(7):1010428320938492. 10.1177/1010428320938492 32635826

[27] Hinshaw DC Shevde LA. The Tumor Microenvironment Innately Modulates Cancer Progression. Cancer Res (2019) 79(18):4557–66. 10.1158/0008-5472.CAN-18-3962 PMC6744958 31350295

[28] Salem ME Weinberg BA Xiu J El-Deiry WS Hwang JJ Gatalica Z. Comparative Molecular Analyses of Left-Sided Colon, Right-Sided Colon, and Rectal Cancers. Oncotarget (2017) 8(49):86356–68. 10.18632/oncotarget.21169 PMC5689690 29156800

[29] Canisius S Martens JW Wessels LF. A Novel Independence Test for Somatic Alterations in Cancer Shows That Biology Drives Mutual Exclusivity But Chance Explains Most Co-Occurrence. Genome Biol (2016) 17(1):261. 10.1186/s13059-016-1114-x 27986087 PMC5162102

[30] Xu M Xu X Pan B Chen X Lin K Zeng K. Lncrna SATB2-AS1 Inhibits Tumor Metastasis and Affects the Tumor Immune Cell Microenvironment in Colorectal Cancer by Regulating Satb2. Mol Cancer (2019) 18(1):135. 10.1186/s12943-019-1063-6 31492160 PMC6729021

[31] Friedman RC Farh KK Burge CB Bartel DP. Most Mammalian mRNAs Are Conserved Targets of MicroRNAs. Genome Res (2009) 19(1):92–105. 10.1101/gr.082701.108 18955434 PMC2612969

[32] Bao S Hu T Liu J Su J Sun J Ming Y. Genomic Instability-Derived Plasma Extracellular Vesicle-Microrna Signature as a Minimally Invasive Predictor of Risk and Unfavorable Prognosis in Breast Cancer. J Nanobiotechnol (2021) 19(1):22. 10.1186/s12951-020-00767-3 PMC7802300 33436002

[33] Cai W Xu Y Yin J Zuo W Su Z. Mir-552-5p Facilitates Osteosarcoma Cell Proliferation and Metastasis by Targeting Wif1. Exp Ther Med (2019) 17(5):3781–8. 10.3892/etm.2019.7361 PMC6447863 30988764

[34] Shi X Li Y Sun Y Zhao X Sun X Gong T. Genome-Wide Analysis of lncRNAs, miRNAs, and Mrnas Forming a Prognostic Scoring System in Esophageal Squamous Cell Carcinoma. PeerJ (2020) 8:e8368. 10.7717/peerj.8368 32095316 PMC7017795

[35] John B Enright AJ Aravin A Tuschl T Sander C Marks DS. Human MicroRNA Targets. PloS Biol (2004) 2(11):e363. 10.1371/journal.pbio.0020363 15502875 PMC521178

[36] Chen Y Wang X. Mirdb: An Online Database for Prediction of Functional microRNA Targets. Nucleic Acids Res (2020) 48(D1):D127–31. 10.1093/nar/gkz757 PMC6943051 31504780

[37] Hua M Li J Wang C Shao L Hou M Peng J. Aberrant Expression of microRNA in CD4(+) Cells Contributes to Th17/Treg Imbalance in Primary Immune Thrombocytopenia. Thromb Res (2019) 177:70–8. 10.1016/j.thromres.2019.03.005 30856381

[38] Ashizawa M Okayama H Ishigame T Thar Min AK Saito K Ujiie D. Mirna-148a-3p Regulates Immunosuppression in DNA Mismatch Repair-Deficient Colorectal Cancer by Targeting PD-L1. Mol Cancer Res MCR (2019) 17(6):1403–13. 10.1158/1541-7786.MCR-18-0831 30872332

[39] Gonzalez-Martin A Adams BD Lai M Shepherd J Salvador-Bernaldez M Salvador JM. The microRNA Mir-148a Functions as a Critical Regulator of B Cell Tolerance and Autoimmunity. Nat Immunol (2016) 17(4):433–40. 10.1038/ni.3385 PMC4803625 26901150

[40] Koch A Joosten SC Feng Z de Ruijter TC Draht MX Melotte V. Analysis of DNA Methylation in Cancer: Location Revisited. Nat Rev Clin Oncol (2018) 15(7):459–66. 10.1038/s41571-018-0004-4 29666440

[41] Parry A Rulands S Reik W. Active Turnover of DNA Methylation During Cell Fate Decisions. Nat Rev Genet (2021) 22(1):59–66. 10.1038/s41576-020-00287-8 33024290

[42] Bond CE Liu C Kawamata F McKeone DM Fernando W Jamieson S. Oncogenic BRAF Mutation Induces DNA Methylation Changes in a Murine Model for Human Serrated Colorectal Neoplasia. Epigenetics (2018) 13(1):40–8. 10.1080/15592294.2017.1411446 PMC5836984 29235923

[43] Yang SY Cho MS Kim NK. Difference Between Right-Sided and Left-Sided Colorectal Cancers: From Embryology to Molecular Subtype. Expert Rev Anticancer Ther (2018) 18(4):351–8. 10.1080/14737140.2018.1442217 29458272

[44] Sillo TO Beggs AD Morton DG Middleton G. Mechanisms of Immunogenicity in Colorectal Cancer. Br J Surg (2019) 106(10):1283–97. 10.1002/bjs.11204 PMC6772007 31216061

[45] Cai W Zhou D Wu W Tan WL Wang J Zhou C. Mhc Class Ii Restricted Neoantigen Peptides Predicted by Clonal Mutation Analysis in Lung Adenocarcinoma Patients: Implications on Prognostic Immunological Biomarker and Vaccine Design. BMC Genomics (2018) 19(1):582. 10.1186/s12864-018-4958-5 30075702 PMC6090856

[46] Thakur P Dadsetan S Fomina AF. Bidirectional Coupling Between Ryanodine Receptors and Ca2+ Release-Activated Ca2+ (Crac) Channel Machinery Sustains Store-Operated Ca2+ Entry in Human T Lymphocytes. J Biol Chem (2012) 287(44):37233–44. 10.1074/jbc.M112.398974 PMC3481322 22948152

[47] Fanelli GN Dal Pozzo CA Depetris I Schirripa M Brignola S Biason P. The Heterogeneous Clinical and Pathological Landscapes of Metastatic Braf-Mutated Colorectal Cancer. Cancer Cell Int (2020) 20:30. 10.1186/s12935-020-1117-2 32015690 PMC6990491

[48] Menyhart O Kakisaka T Pongor LS Uetake H Goel A Gyorffy B. Uncovering Potential Therapeutic Targets in Colorectal Cancer by Deciphering Mutational Status and Expression of Druggable Oncogenes. Cancers (2019) 11(7):983. 10.3390/cancers11070983 PMC6679198 31337155

[49] Liang J Tian C Zeng Y Yang Q Liu Y Liu Y. Foxa1(+) Regulatory T Cells: A Novel T Cell Subset That Suppresses Antitumor Immunity in Lung Cancer. Biochem Biophys Res Commun (2019) 514(1):308–15. 10.1016/j.bbrc.2019.04.152 31036318

[50] Yi M Xu L Jiao Y Luo S Li A Wu K. The Role of Cancer-Derived microRNAs in Cancer Immune Escape. J Hematol Oncol (2020) 13(1):25. 10.1186/s13045-020-00848-8 32222150 PMC7103070

[51] Cheng YW Pincas H Bacolod MD Schemmann G Giardina SF Huang J. Cpg Island Methylator Phenotype Associates With Low-Degree Chromosomal Abnormalities in Colorectal Cancer. Clin Cancer Res an Off J Am Assoc Cancer Res (2008) 14(19):6005–13. 10.1158/1078-0432.CCR-08-0216 PMC3268558 18829479

[52] Wang F Zhao Q Wang YN Jin Y He MM Liu ZX. Evaluation of POLE and POLD1 Mutations as Biomarkers for Immunotherapy Outcomes Across Multiple Cancer Types. JAMA Oncol (2019) 5(10):1504–6. 10.1001/jamaoncol.2019.2963 PMC6696731 31415061

