# Supplementary material for: Regulatory Network Analysis of Mutated Genes Based on Multi-Omics Data Reveals the Exclusive Features in Tumor Immune Microenvironment Between Left-Sided and Right-Sided Colon Cancer
Source: Front Oncol. 2021 Jun 15;11:685515. doi: 10.3389/fonc.2021.685515 (PMC8239301; doi:10.3389/fonc.2021.685515)
Supplement: Supplementary file 1 [file Table_1.docx]

**Supplementary Figures**


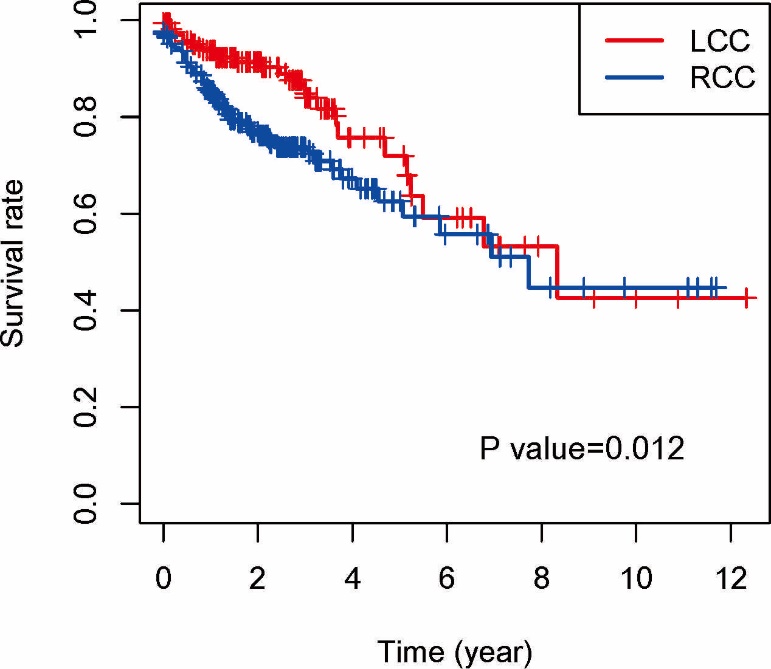


**Figure S1.** Comparison of survival curves for LCC and RCC patients by Kaplan-Meier analyses.


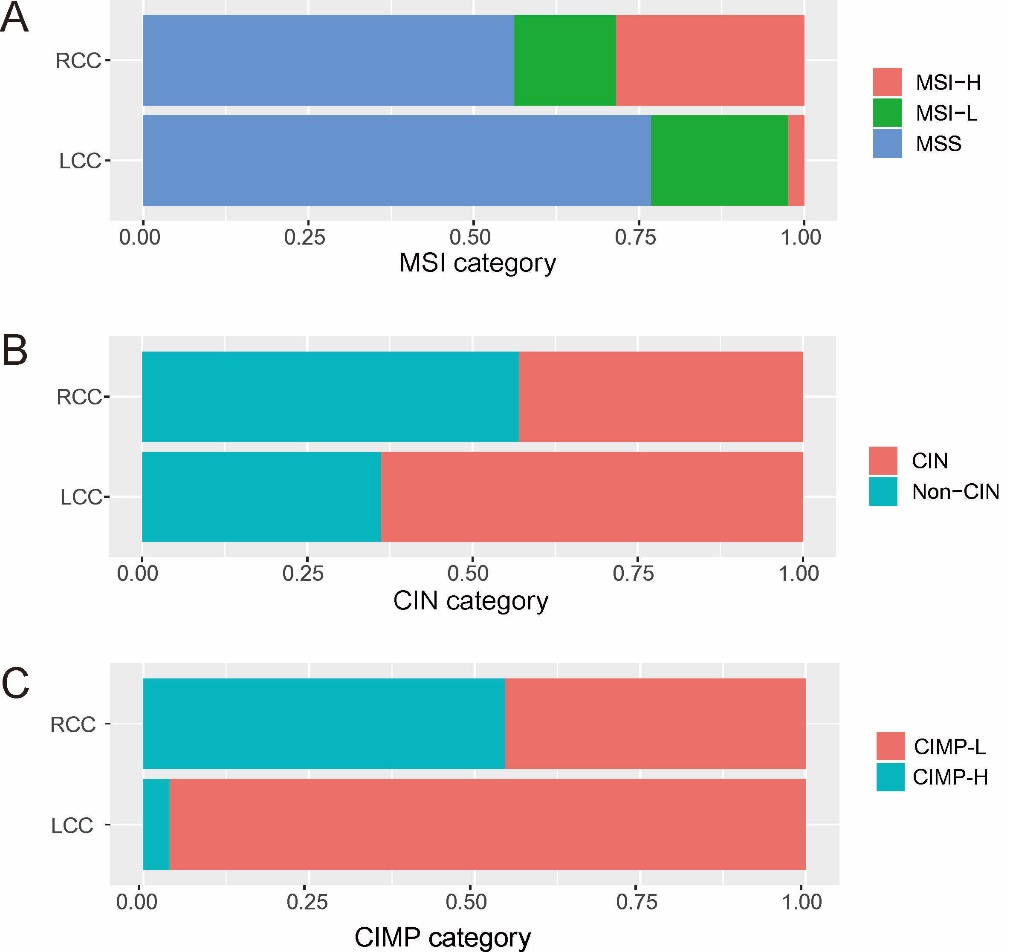


**Figure S2.** Comparison of MSI (A), CIN (B) and CIMP(C) subtypes distribution between LCC and RCC.


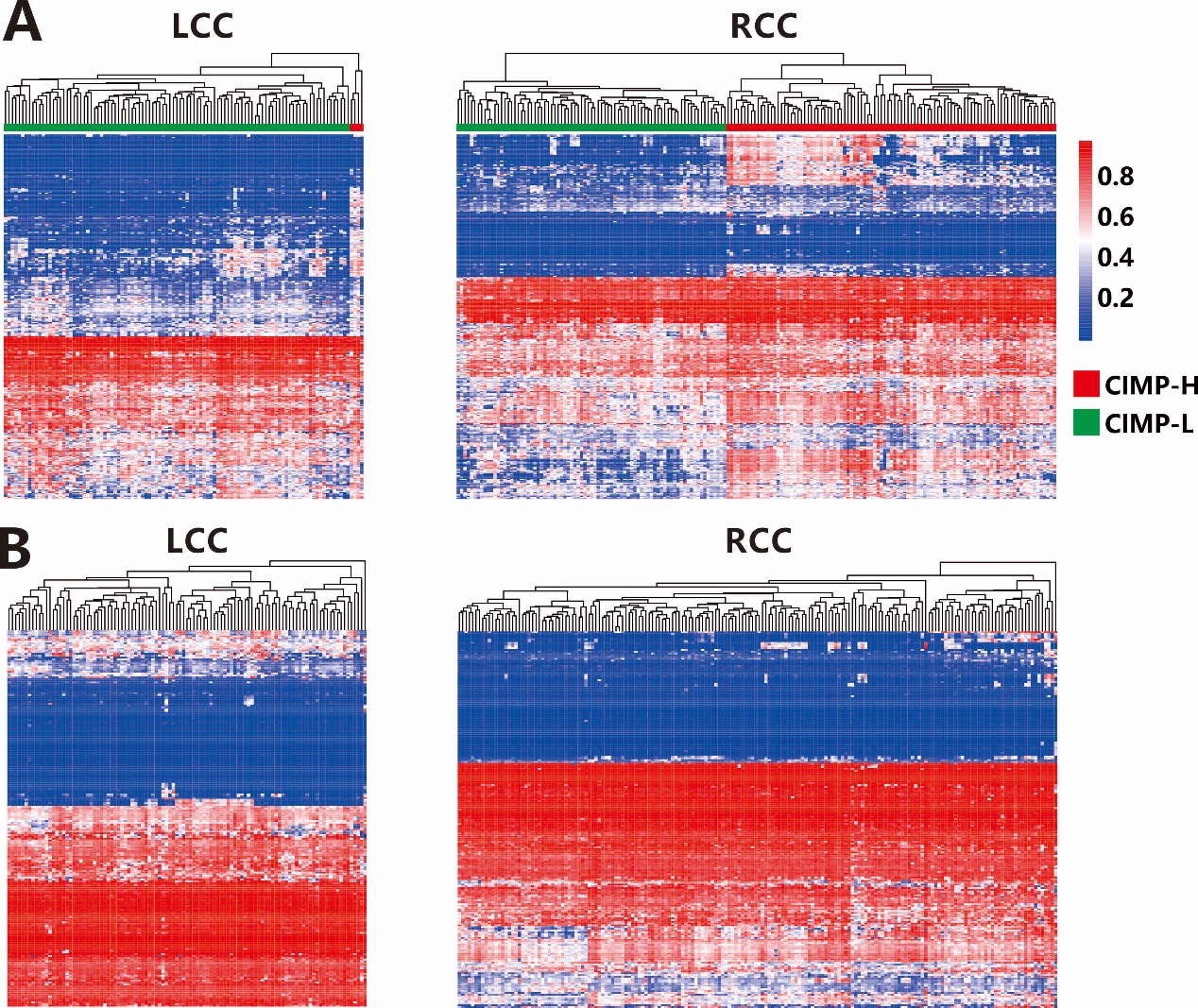


**Figure S3.** The result of the unsupervised consensus clustering analysis in LCC and RCC based on the DNA methylation levels of the 5 markers (A) and 7 methylation-related genes (B).


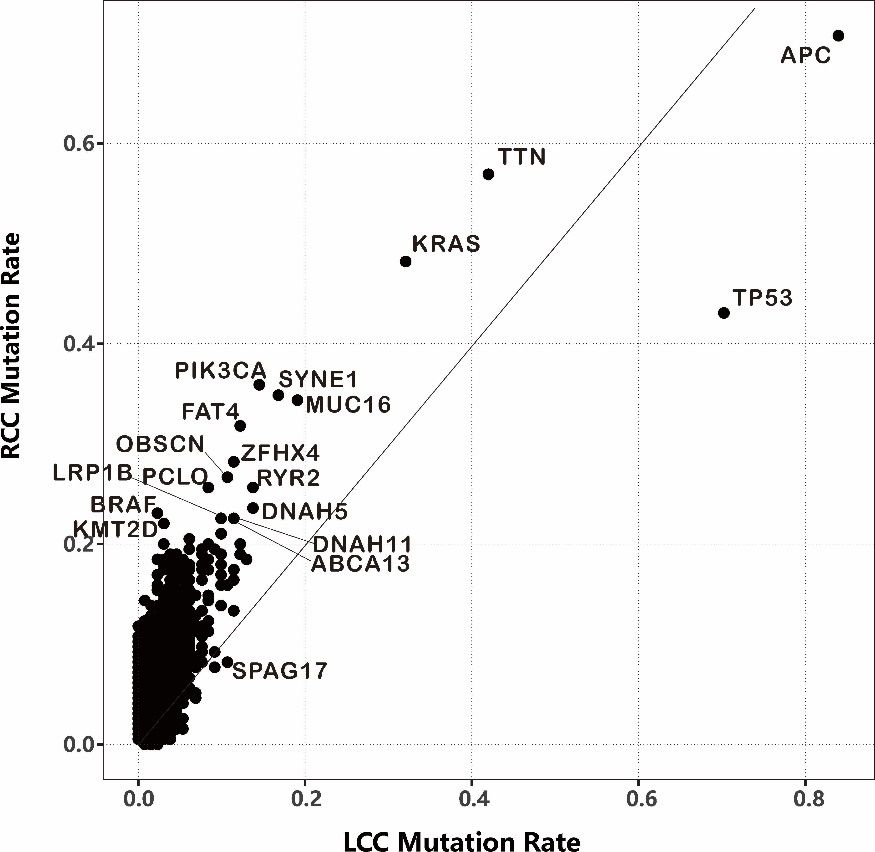


**Figure S4.** Mutation rate of genes in LCC and RCC.


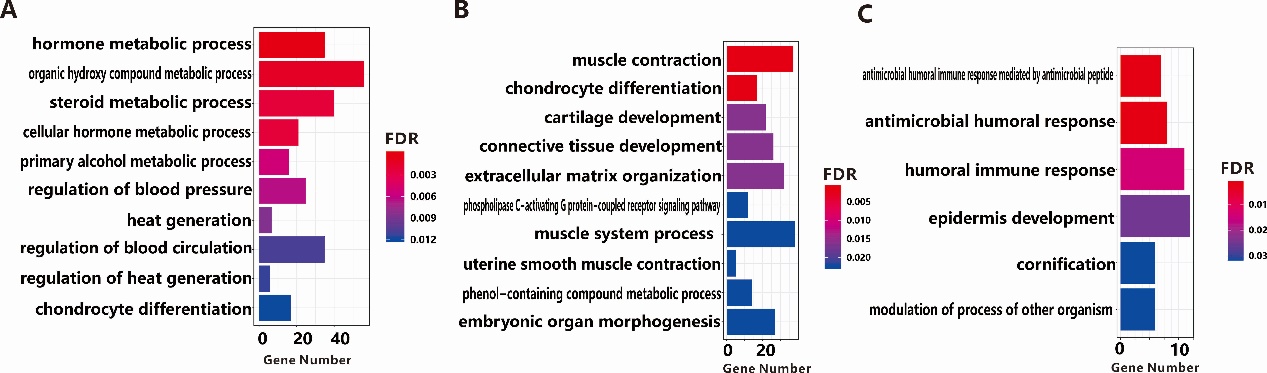


**Figure S5. (**A) GO functional enrichment analysis of TMGs-downregulating DEGs in RCC. (B) GO functional enrichment analysis of TMGs-downregulating DEGs in LCC. (C) GO functional enrichment analysis of TMGs-downregulating DEGs (top 1000 FC) in LCC.


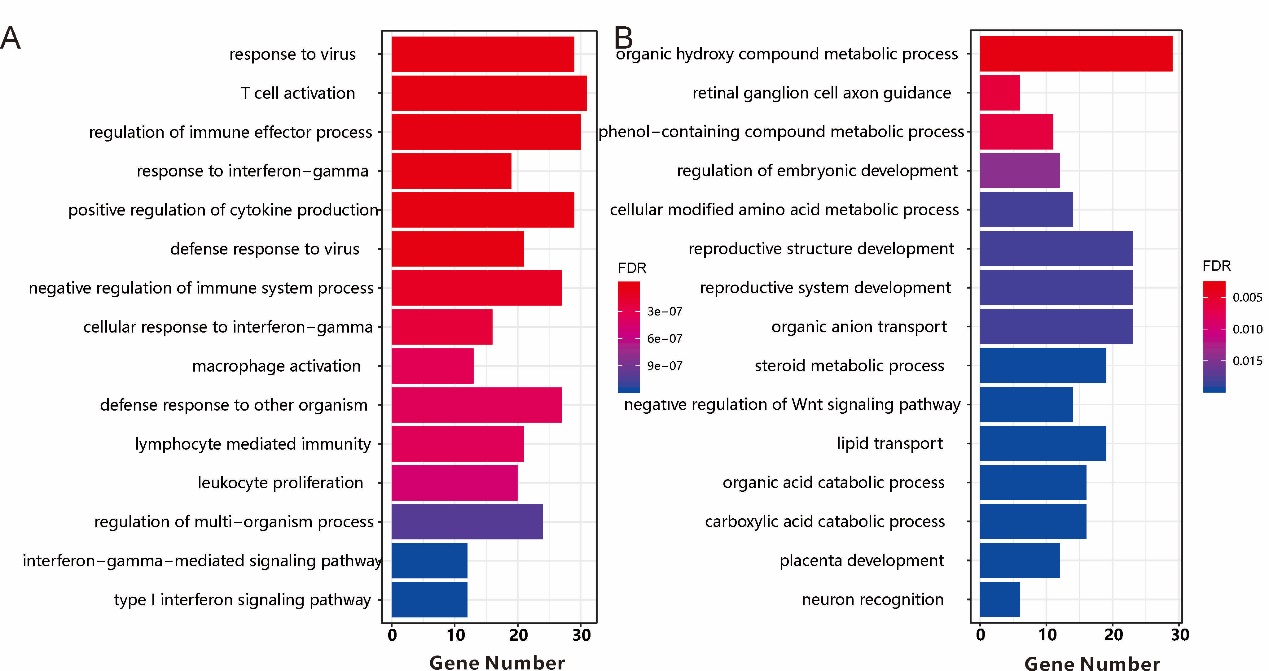


**Figure S6. (**A) GO functional enrichment analysis of *BRAF*-upregulating DEGs in RCC. (B) GO functional enrichment analysis of *BRAF*-downregulating DEGs in RCC.


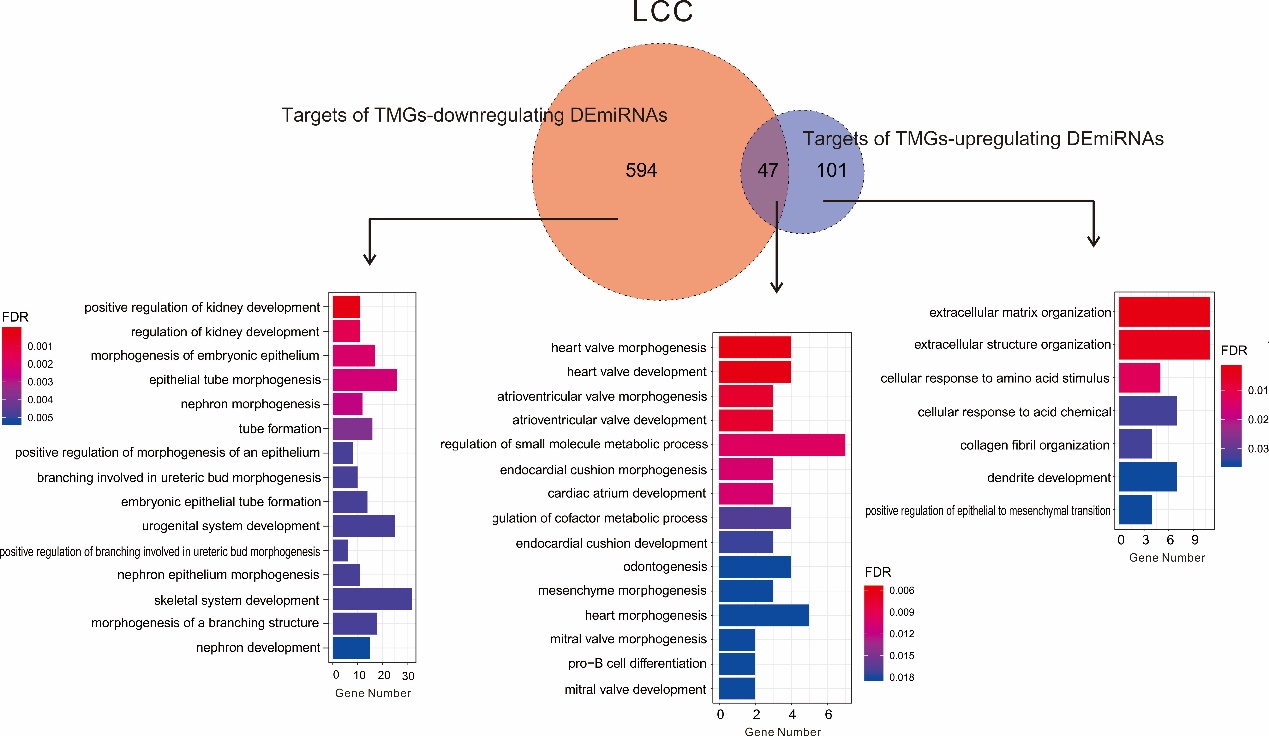


**Figure S7.** Venn diagrams show the overlap between the targets of TMG-downregulating DEmiRNAs and the targets of TMG-upregulating DEmiRNAs in LCC. The bar plots show the GO functional enrichment analysis of the corresponding parts of genes in the Venn diagram.


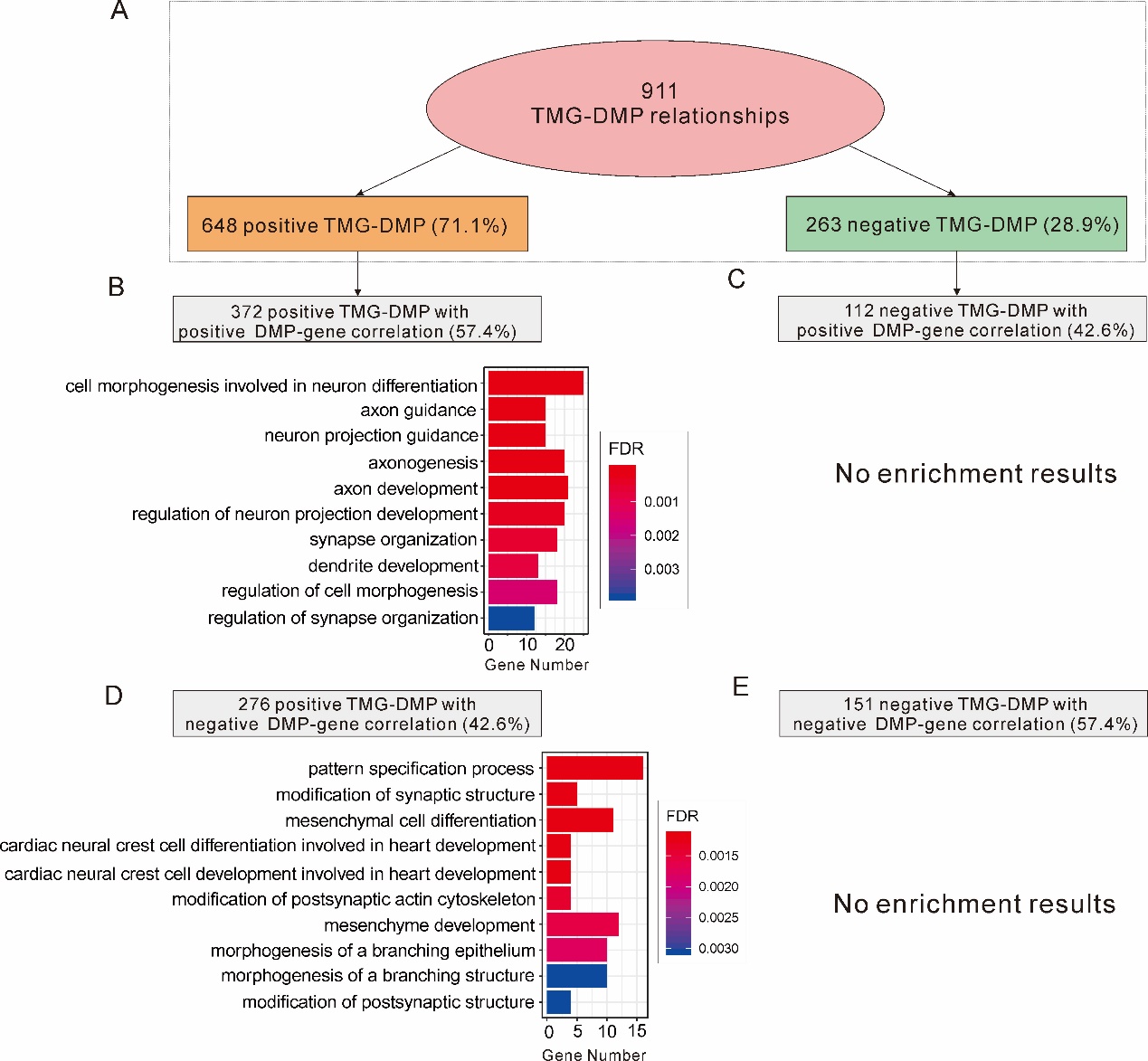


**Figure S8.** Relationships between immune infiltration and DMPs regulated by TMGs in LCC. (A) The number and the proportions of positive and negative TMG-DMP relationships in LCC. (B) The GO functional enrichment analysis of the genes which are positively correlated with the DNA methylation level of TMGs-upregulating DMPs in LCC. (C) No significant result was found in the genes which are positively correlated with the DNA methylation level of TMGs-downregulating DMPs in LCC. (D) The GO functional enrichment analysis of the genes which are negatively correlated with the DNA methylation level of TMGs-upregulating DMPs in LCC. (E) No significant result was found in the genes which are negatively correlated with the DNA methylation level of TMGs-downregulating DMPs in LCC.


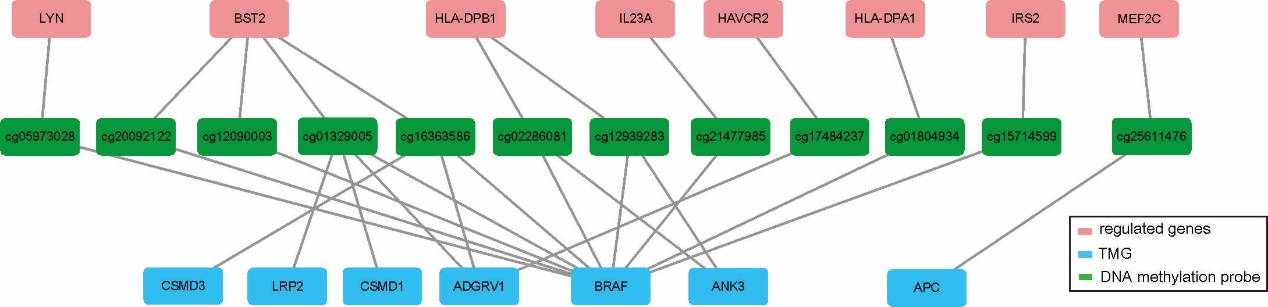


**Figure S9.** The network among TMGs (bule), DNA methylation probes (green) and gens (red) whose functions are annotated with positive regulation of leukocyte proliferation.


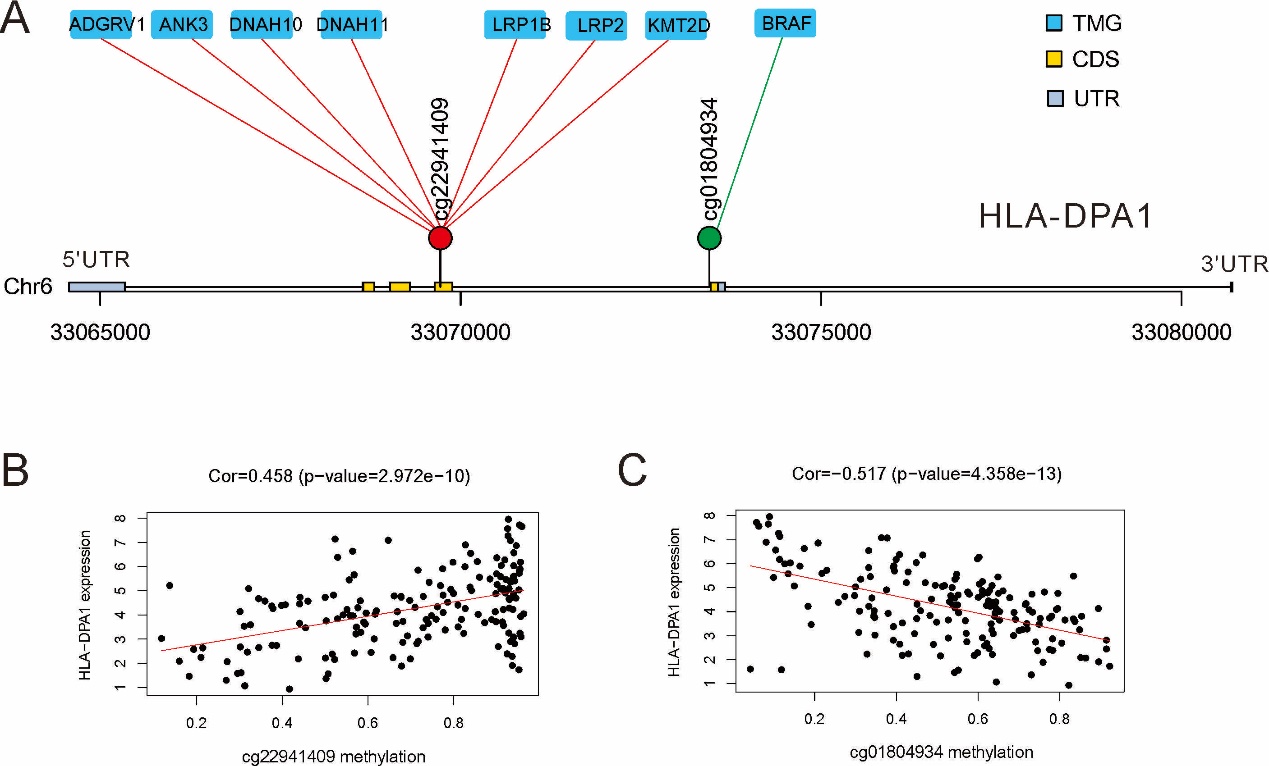


**Figure S10.** (A) The relationships between TMGs and methylation probes of *HLA-DPA1*. (B) The DNA methylation level of probe cg22941409 is positively correlated with the expression level of *HLA-DPA1*. (C) The DNA methylation level of the probe cg01804934 is negatively correlated with the expression level of *HLA-DPA1*.


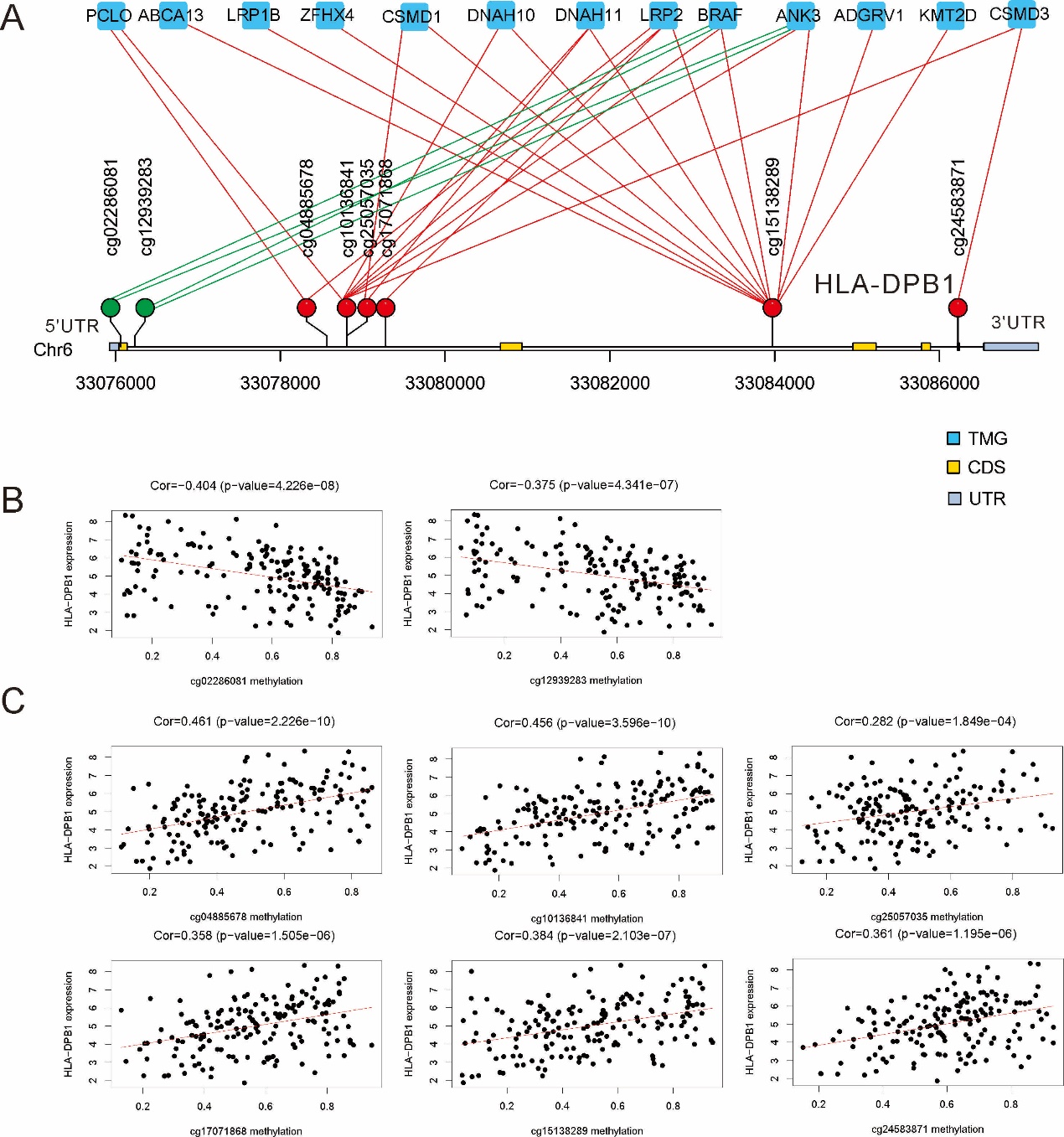


**Figure S11** (A) The relationships between TMGs and methylation probes of *HLA-DPB1*. (B) The DNA methylation levels of probes cg02286081 and cg12939283 were negatively correlated with the expression level of *HLA-DPB1*. (C) The DNA methylation levels of probes cg04885678, cg10136841, cg25057035, cg17071868, cg15138289, and cg24583871 were positively correlated with the expression level of *HLA-DPB1*.
